# Beta Spike-Presenting SARS-CoV-2 Virus-like Particle Vaccine Confers Broad Protection against Other VOCs in Mice

**DOI:** 10.3390/vaccines12091007

**Published:** 2024-09-02

**Authors:** Irfan Ullah, Kelly Symmes, Kadiatou Keita, Li Zhu, Michael W. Grunst, Wenwei Li, Walther Mothes, Priti Kumar, Pradeep D. Uchil

**Affiliations:** 1 Section of Infectious Diseases, Department of Internal Medicine, Yale University School of Medicine, New Haven, CT 06520, USA; irfan.ullah@yale.edu (I.U.); kelly.symmes@yale.edu (K.S.); li.zhu@yale.edu (L.Z.); priti.kumar@yale.edu (P.K.); 2 Department of Microbial Pathogenesis, Yale University School of Medicine, New Haven, CT 06510, USA; k.keita@yale.edu (K.K.); mikey.grunst@yale.edu (M.W.G.); wenwei.li@yale.edu (W.L.); walther.mothes@yale.edu (W.M.)

**Keywords:** SARS-CoV-2, vaccine, virus-like particles, intranasal, intramuscular, neutralizing antibodies, variants of concern, cross-VOC protection, Beta, Omicron

## Abstract

Virus-like particles (VLPs) are non-infectious and serve as promising vaccine platforms because they mimic the membrane-embedded conformations of fusion glycoproteins on native viruses. Here, we employed SARS-CoV-2 VLPs (SMEN) presenting ancestral, Beta, or Omicron spikes to identify the variant spike that elicits potent and cross-protective immune responses in the highly sensitive K18-hACE2 challenge mouse model. A combined intranasal and intramuscular SMEN vaccine regimen generated the most effective immune responses to significantly reduce disease burden. Protection was primarily mediated by antibodies, with minor but distinct contributions from T cells in reducing virus spread and inflammation. Immunization with SMEN carrying ancestral spike resulted in 100, 75, or 0% protection against ancestral, Delta, or Beta variant-induced mortality, respectively. However, SMEN with an Omicron spike provided only limited protection against ancestral (50%), Delta (0%), and Beta (25%) challenges. By contrast, SMEN with Beta spikes offered 100% protection against the variants used in this study. Thus, the Beta variant not only overcame the immunity produced by other variants, but the Beta spike also elicited diverse and effective humoral immune responses. Our findings suggest that leveraging the Beta variant spike protein can enhance SARS-CoV-2 immunity, potentially leading to a more comprehensive vaccine against emerging variants.

## 1. Introduction

The SARS-CoV-2 Spike protein (S) present on the surface of virions is required for engaging the receptor ACE2 to enter cells [1,2,3,4,5]. Neutralizing humoral immune responses directed towards the S protein effectively block virus entry and have been correlated with protection from severe disease [4,6]. Several vaccine platforms developed during the COVID-19 pandemic have incorporated various forms of S as target antigens [7,8,9]. Among these, mRNA-based vaccines emerged as the preferred platform in the USA because of their high potency, cost-effectiveness in manufacturing, and rapid large-scale deployment capabilities, which addressed a significant challenge in vaccine development, particularly during the COVID-19 pandemic [10]. Although the efficacy of most vaccines to prevent SARS-CoV-2 infection and transmission has been reduced against variants of concern (VOCs), vaccine-based immunity remains protective against severe disease and hospitalization [11]. In recent years, several studies have highlighted rare but noteworthy adverse effects associated with mRNA vaccines, including cardiovascular complications, thrombosis, thrombocytopenia, and fatigue, which have fueled vaccine hesitancy [12,13,14,15,16,17]. Other side effects, such as Bell’s palsy and transverse myelitis, have been observed in individuals receiving inactivated virus-based vaccines [18]. In addition, adenoviral vector-based vaccines were found to trigger vaccine-induced immune thrombocytopenia and thrombosis (VITT) due to cross-reactive antibodies that target platelet factor 4 [19]. Consequently, the continued development of different vaccine platforms, including traditional protein-based vaccines, is essential for providing additional choices for individuals with comorbidities and safety concerns to improve acceptance rates.

Virus-like particles are a viable vaccine platform that has been evaluated for effectiveness in human clinical trials against respiratory viruses, including influenza and respiratory syncytial virus (RSV). Notably, CoV-2 VLP-based vaccines have also been assessed in several clinical trials against SARS-CoV-2, highlighting its potential [20,21]. SARS-CoV-2 virus-like particles (VLPs; SMEN) can be readily generated without any need for proprietary technology by expressing four SARS-CoV-2 structural proteins in cell culture: spike (S), membrane (M), envelope (E), and nucleocapsid (N). Unlike mRNA vaccines that require below-freezing temperatures, SMEN VLPs are stable for weeks at 4 °C [22,23]. They are non-infectious as they do not contain any replication-competent genetic material. The high immunogenicity of SMEN particles makes them suitable for a variety of vaccine efficacy experiments and are valuable as research tools [24,25,26]. VLPs have been used to study the impact of mutations in structural proteins and to screen therapeutics [27]. Importantly, their modular nature allows easy adaptation to S proteins from emerging variants, facilitating studies that examine their breadth and protective efficacy. SMEN VLPs can be administered via the traditional intramuscular route to elicit peripheral immunity and control the systemic spread of the virus. In addition, SMEN VLPs can be safely administered intranasally to potentially induce effective immunity in the respiratory mucosa, which is crucial for preventing virus transmission and remains a broad goal in vaccinology [28] Unlike mRNA or adenovirus-based vaccine platforms, SMEN particles mimic the membrane-embedded conformations of the spike protein on native viruses [29]. Furthermore, the inclusion of additional viral proteins (M, E, and N) in SMEN particles can augment immunity by generating responses to a broad range of viral proteins [10,30,31,32].

Neutralizing antibodies contribute significantly towards protection against SARS-CoV-2 infection [33]. Therefore, fundamental investigations comparing the cross-protective neutralizing responses elicited by S proteins from prominent variants of concern (VOCs) are essential for guiding future vaccine development. Additionally, it is crucial to determine whether the elicited humoral immune responses correlate with protection against homologous and heterologous VOCs in vivo using stringent challenge models. In this study, we employed the SMEN VLP vaccine to assess the efficacy of S protein derived from ancestral, Delta, Beta, and Omicron variants in eliciting robust cross-protective immune responses in the highly sensitive K18-hACE2 mouse challenge model. Our data indicate that a combined intranasal and intramuscular administration regimen of the SMEN vaccine generates the most effective immune response for the virologic control and mitigation of lung inflammation. Protection was largely mediated by antibodies, with T cells contributing towards limiting virus dissemination and inflammation. Notably, the Beta variant not only overcame the immunity produced by other variants, but SMEN bearing S from the Beta VOC also elicited the most diverse and effective humoral immune response. Our results indicate that harnessing the S protein from the Beta variant may contribute to a more comprehensive vaccine targeting emerging variants.

## 2. Materials and Methods

### 2.1. Ethics Statement

Animal experiments were approved by the Yale Institutional Biosafety Committee (IBC) and Institutional Animal Care and Use Committee (IACUC). Protocols for imaging SARS-CoV-2 infected animals using IVIS (PerkinElmer, Inc., Waltham, MA, USA) under Animal Biosafety Level 3 (ABSL-3) conditions were approved by the IACUC, IBSCYU, and Yale Animal Resources Center (YARC). Anesthesia was administered to the animals during procedures such as virus inoculation, blood collection, and imaging to minimize their pain and discomfort.

### 2.2. Cell and Viruses

The American Type Culture Collection (ATCC) provided the Vero E6 (CRL-1586) human embryonic kidney (HEK-293) cells, which were grown at 37 °C in a RPMI medium. This medium was supplemented with 10% fetal bovine serum (FBS), 10 mM HEPES (pH 7.3), 1 mM sodium pyruvate, 1× non-essential amino acids, and 100 U/mL of penicillin–streptomycin. SARS-CoV-2 (WA1) strain expressing NanoLuc luciferase (nLuc) was procured from Craig Wilen at Yale University. The B.1.617.2 (Delta), B.1.351 (Beta), and Omicron (BA.1) variants of concern (VOC) were isolated from a Yale New Haven Hospital patient and obtained through Craig Wilen. All viral strains were verified using next-generation sequencing at the Yale Center for Genomic Analysis. We generated NanoLuc luciferase (nLuc)-expressing reporter viruses for the Delta, Beta, and Omicron VOC using the circular polymerization extension reaction (CPER) method, as previously described [34,35,36]. The described reporter viruses were employed for non-invasive bioluminescence imaging (BLI) of infected mice. The process involved converting viral RNA to cDNA using a PrimeScript RT kit (Takara Bio Inc, San Jose, CA, USA) with a combination of random and oligo dT primers. This cDNA served as a template for amplifying 11 overlapping fragments, approximately 2–3 kb in length, using specific primers and PrimeStar GXL polymerase (Takara Bio Inc., San Jose, CA, USA). A nLuc-P2A cassette was inserted into the N-terminus of the nucleocapsid gene. The purified overlapping fragments were then circularized through CPER, utilizing a Hepatitis Delta virus ribozyme-spacer-CMV promoter cassette that overlapped the genome at 3′ and 5′ ends. CPER products were introduced into HEK293 cells via polyethyleneimine transfection and cultured under BSL3 conditions. These cells were subsequently resuspended and co-cultured with Vero E6 ACE2/TMPRSS2 (Vero AT) (BEI resources, Manassas, VA, USA) cells for 5–9 days until cytopathic effects (CPE) were evident and nLuc activity was detected in the culture supernatants. Viruses were propagated in Vero AT cells, infected in T150 cm^2^ flasks at a multiplicity of infection (MOI) of 0.1. Culture supernatants were collected after 18–24 h when CPE was clearly visible. Cell debris was removed through sedimentation and filtration to generate virus stocks. The viruses were concentrated using PEG-it Virus Precipitation Solution (System Biosciences, LLC, Palo Alto, CA, USA), mixed with the virus-containing supernatant according to manufacturer instructions, and incubated overnight at 4 °C. The precipitated virus was then harvested by centrifugation, resuspended in PBS, and stored at −80 °C in aliquots. All work involving infectious SARS-CoV-2 was conducted in Institutional Biosafety Committee-approved BSL3 and A-BSL3 facilities at the Yale University School of Medicine [34,35,36].

### 2.3. Mouse Experiments

The Yale University Animal Resource Centre (YARC) housed all animals in its specific pathogen-free (SPF) barrier facility, following a 14:10 light-dark cycle. Separate rooms were used for breeding populations and infected animals. SARS-CoV-2-infected animals were kept in a BSL3 containment room. Yale University Environmental Health Services guidelines were followed for the disposal and decontamination of cages, animal waste, bedding, and carcasses. Personnel handling replication-competent virus-infected animals in ABSL3 conditions wore protective gear, including pressurized air-purified respirators, double gloves, shoe and sleeve covers, and disposable gowns. The Jackson Laboratory (Bar Harbor, ME, USA) provided heterozygous hACE2 transgenic B6 mice. Experiments utilized 6–8-week-old male and female mice. Heterozygous mice were bred and genotyped using recommended primer sets. Cohort sizes ranged from 4 to 8 animals, with two to three biological replicates to enable statistical testing and parallel evaluation. The number of animals per cohort was determined through a priori power analysis based on pilot experiments and previous studies’ data. [37,38,39,40]. Experiments were conducted using randomly chosen animals from sex- and age-matched littermates. No animals were removed from the study due to illness following the experimental procedures. Efforts were made to include equal numbers of male and female mice in the experiments whenever possible to ensure that sex did not become a confounding biological factor during data analysis.

### 2.4. Generation of SMEN VLPs

SMEN VLPs were prepared by transfecting HEK293 cells at 60% confluence with individual plasmids expressing SARS-CoV-2 spike (S), membrane (M), envelope (E), and nucleocapsid (N) proteins at a ratio of 1:1:5:5 using polyethyleneimine (PEI, Polysciences, Inc, Warrington, PA, USA) as a transfection reagent. Transfected cells were washed 6 h post-transfection and replenished with RPMI supplemented with 10% FBS. The culture supernatant was collected 48 h post-infection, cleared through a 0.45 µm filter, and partially purified by ultracentrifugation after being overlaid with 15% sucrose, followed by storage at −80 °C in aliquots. The SMEN VLPs protein was estimated using Pierce^TM^ BCA protein assay kit (ThermoFisher, Waltham, MA, USA, Cat# 23225) and further characterized by western blot and cryo-ET analyses (see below).

### 2.5. Western Blot

To test the incorporation of individual proteins, partially purified SMEN VLPs presenting different spike proteins were solubilized in 1X NuPAGE sample buffer, denatured, and subjected to SDS-PAGE analyses using NuPAGE 4–12% Bis-Tris gels (Thermo Fisher Scientific, Inc., Waltham, MA, USA). After transfer, the membranes were probed with antibodies to the SARS-CoV-2 spike, nucleocapsid, membrane, and envelope proteins (Cell Signaling Technology, 1:2000). To test the ability of sera from SMEN-vaccinated mice to recognize S, M, E, and N proteins, SMEN VLPs with different spikes were processed as described above and probed with mouse sera at a 1:2000 dilution. The membranes were probed with appropriate secondary antibodies (1:10,000) conjugated to horseradish peroxidase (HRP), developed using SuperSignal™ West Pico PLUS Chemiluminescent Substrate (Thermo Fisher Scientific, Inc., Waltham, MA, USA), and images were acquired using Amersham ImageQuant™ 800 Western blot imaging systems (Cytiva, Marlborough, MA, USA).

### 2.6. SMEN VLPs Vaccination Regimen and SARS-CoV-2 Challenge

For all in vivo experiments, 6 to 8 weeks male and female mice were used. K18-hACE2 transgenic mice (heterozygous) were administered intranasally (i.n.; mucosal vaccination) or intramuscularly (i.m.; systemic vaccination) with a mix of SMEN VLPs (250 µg) and vaccigrade TLR7/TLR8 agonist R848 (30 µg, vaccigrade was used only i.m.) (InvivoGen US, San Diego, CA, USA) as an adjuvant at each site. Uninfected mice or mice administered R848 alone were used as controls. Intranasal administration was performed under anesthesia at a volume of 25–30 µL. The mice were boosted once with the same amount of VLPs, i.n. and i.m., 14 days after the first challenge. Blood was collected on day 21 to determine antibody titers. The anesthetized mice were challenged intranasally to SARS-CoV-2-nLuc viruses, receiving 1 × 10^5^ PFU in a 25–30 µL solution. Anesthesia was administered using 0.5–5% isoflurane, delivered via a precision Dräger vaporizer with oxygen flowing at 1 L/min. Initial body weight was recorded as 100%. For mortality studies, mice were observed every 8–12 h beginning six days post-viral challenge. Mice exhibiting lethargy, severe illness, or weight loss exceeding 20% of their starting body weight were euthanized and recorded as fatalities in Kaplan-Meier survival analyses. Recovery was defined as regaining all previously lost weight.

### 2.7. Passive Transfer of Sera

C57BL/6J mice (Jackson Laboratory, Bar Harbor, ME, USA) were vaccinated with SMEN_WA1_, as described above. A total of 21 days after the initial vaccination, the mice were anesthetized using isoflurane inhalation (3–5% isoflurane, oxygen flow rate of 1.5 L/min), and blood was collected from either the eye or submandibular vein. Serum was obtained from whole blood by sedimenting at 2000× *g* for 15 min at room temperature. Sera from mice before vaccination (pre-immune sera) or after vaccination (immune sera) was combined, and 500 µL of this serum was administered intraperitoneally to naïve K18-hACE2 mice (B6 background) one day prior to SARS-CoV-2 infection.

### 2.8. Immunodepletion of CD4^+^ and CD8^+^ T Cells

To investigate the contribution of T cells to vaccine-mediated protection in mice, CD4^+^ T and CD8^+^ T cells were depleted using mouse anti-CD4 (BioX-Cell, Lebanon, NH, USA; clone YTS 191; 12.5 mg/kg body weight) and anti-CD8α (BioX-Cell, Lebanon, NH, USA; clone 53.6.7; 12.5 mg/kg body weight) monoclonal antibodies (mAbs). The mAbs were administered to mice via intraperitoneal (i.p.) injection every two days, starting from −2 days until the experimental endpoint. Rat IgG2b (BioX-Cell, Lebanon, NH USA; 12.5 mg/kg body weight) was used as a control. Blood samples were collected every 48 h via retroorbital bleeding to isolate peripheral blood mononuclear cells (PBMCs) using the Ficoll method. The cells were then stained with antibodies (BioLegend, San Diego, CA, USA) to mouse CD45 (Alexa Fluor™ 488, clone 30-F11), CD3 (Alexa Fluor™ 647, clone 145-2C11), CD4 (PE-Cy7, clone GK1.5), and CD8 (PE, clone 53-6.7), and samples were acquired using a BD Accuri C6 flow cytometer (BD Biosciences, Franklin Lakes, NJ, USA) and C6 sampler v1.0.264.21 software (BD Biosciences, NJ, USA).

### 2.9. Bioluminescence Imaging (BLI) of SARS-CoV-2 Infection

The IVIS imaging protocols for examining SARS-CoV-2-infected animals in ABSL-3 conditions were approved by the Institutional Animal Care and Use Committee, Institutional Biosafety Committee, and Yale Animal Research Committee. The IVIS Spectrum^®^ and XIC-3 animal isolation chamber from (PerkinElmer, Inc., Waltham, MA, USA) were employed to image anesthetized mice or individual organs while maintaining biological containment. Mice were anesthetized using isoflurane inhalation (3–5%, oxygen flow 1.5 L/min) before and during bioluminescence imaging (BLI) with the XGI-8 Gas Anesthesia System. Prior to imaging, mice were administered 100 µL of diluted furimazine (1:40 in endotoxin-free PBS; Promega Inc., Madison, WI, USA) retro-orbitally. The animals were then placed in the isoflurane-saturated XIC-3 chamber and imaged dorsally and ventrally on specified days post-infection. Following euthanasia and necropsy, the animals were reimaged after applying 200 µL of substrate to exposed intact organs. Additional diluted furimazine was added to the organs and allowed to soak for 1–2 min before BLI. Image acquisition and analysis were performed using Living Image v4.7.3 software. Auto-exposure settings were used, with preferences set in the order of exposure time, binning, and f/stop. The luminescent f/stop was set to 2, the photographic f/stop was set to 8, and the binning was set to medium. For comparative analyses, images were compiled and processed collectively using consistent luminescent scales. Photon flux was quantified as luminescent radiance (photons/sec/cm^2^/sr). During luminescent threshold selection, signals were deemed background when the minimum threshold resulted in displayed radiance above non-tissue-containing or known uninfected areas.

### 2.10. SARS-CoV-2 Neutralization Assay and Calculation of Cross-Reactive Neutralization Index

Serum samples were collected from immunized mice (21 days post-vaccination, before challenge) and were subjected to heat inactivation at 56 °C for 30 min and then diluted in 4-fold serial dilutions in serum-free RPMI, starting at a dilution of 1:10. Each serum dilution (50 µL) and 200 PFU of virus (50 µL; WA1, Delta, Beta or Omicron viruses expressing nLuc reporter) were combined and incubated at 37 °C for 1 h. Subsequently, a 96-well tissue culture plate with sub-confluent VeroE6 cells was infected for 4 h. The cells were washed three times with serum-free RPMI, followed by the addition of fresh RPMI media supplemented with 10% FBS. After 24 h post-infection, the cells were washed twice more with 1× PBS and lysed in 100 µL of 1× passive lysis buffer. A total of 20 µL of the lysates were transferred to a 96-well solid white plate (Costar Inc., Washington, DC, USA), and nLuc activity was measured using the Tristar multi-well Luminometer (Berthold Technology, Bad Wildbad, Germany) for 2.5 s by adding 20 µL of Nano-Glo^®^ substrate (diluted in 1x PBS at a 1:40 dilution) in nanoluc assay buffer (Promega Inc., Madison, WI, USA). An uninfected monolayer of Vero cells treated identically served as a control to determine basal luciferase activity and obtain normalized relative light units. Cells infected with viruses that were treated with sera from non-vaccinated mice were set to 100%. The data were processed and plotted using GraphPad Prism 8 v8.4.3 to calculate IC50 (50% inhibitory concentration) values. Cross-reactive neutralization index (CRNI) was calculated based on the IC50 values using the following formula:

CRNI = (IC50 of sera for heterologous strain/average IC50 for homologous or reference strain) × 100.

A CRNI value approaching 100% signifies a considerable level of cross-reactivity, implying that the antibodies are equally effective against both strains. Conversely, a value significantly below 100% suggests diminished cross-reactivity, indicating that the antibodies exhibit reduced efficacy against the variant strain compared to the reference strain.

### 2.11. Fc-Signaling Assay and Calculation of Cross-Reactive Fc-Signaling Index

Mouse FcgRIV (Genbank accession number: NM_144559.2) was synthesized and cloned into a lentivirus packaging construct (pGenlenti)(Genscript Piscataway, NJ, USA). Jurkat N-FAT Luciferase (JNL) cells (Signosis, Santa Clara, CA, USA) were transduced with VSV-G decorated lentivirus and grown under puromycin selection to generate the JNL-mFcgRIV for the FcgR signaling assay. VeroE6 cells (1 × 10^4^) were seeded in a 96-well tissue culture plate and infected with WA1, Delta, Beta, and Omicron SARS-CoV-2 variants for 4 h. The levels of infection among all SARS-CoV-2 strains used were normalized based on nLuc signals in infected VeroE6 cells as measured 24 h post-infection (equivalent to ~150–200 PFU). The cells were then washed three times with serum-free RPMI, followed by the addition of 150 µL of fresh RPMI media supplemented with 10% FBS. An amount equivalent to IC_50_ of sera from each immunized mouse (please see Section 2.10 for details of serum collection) was mixed with 2 × 10^4^ JNL-mFcgRIV cells in a final volume of 50 µL and added to mock or virus-infected VeroE6 cells and incubated for 4 h. The cells were lysed with 50 µL of Britelite substrate (Perkin Elmer, Waltham, MA, USA) containing lysis buffer, and luciferase activity was measured after 5 min. Mock-infected VeroE6 cells incubated with JNL-mFcgRIV cells and sera were used as a control to normalize the Fc-signaling activity. The cross-reactive Fc-signaling index (CRFSI) was calculated based on the Fc activity measured using IC50 (neutralizing) equivalent sera for each variant used in our study using the following formula:

CRFSI = (Fc signaling activity in IC50 equivalent sera for heterologous strain/average Fc signaling activity with IC50 equivalent sera for homologous or reference strain) × 100.

### 2.12. Plaque Forming Assay

The quantification of virus stock concentrations was conducted using a conventional plaque assay method. Initially, 4 × 10^5^ Vero-E6 cells were placed in a 12-well plate and incubated for a 24-h period. Subsequently, the cells underwent infection with sequential dilutions of the virus stock, followed by the application of 1 mL of pre-heated 0.6% Avicel (RC-581 FMC BioPolymer) in a complete RPMI medium (Thermo Fisher Scientific, Waltham, MA, USA). Following a 48-h incubation, the cells were treated with 10% paraformaldehyde for 15 min to fix them, then stained using 0.2% crystal violet (Sigma Aldrich, St. Louis, MO, USA) in 20% ethanol (Sigma Aldrich, St. Louis, MO, USA) for 1 h to visualize the plaques. The plates were then washed with water to enhance plaque visibility.

### 2.13. Measurement of Viral Burden

Organs, including the brain and lungs, were collected and weighed from both infected and uninfected mice. These organs were then homogenized in 1 mL of serum-free RPMI medium containing penicillin-streptomycin. The homogenization process utilized a 2 mL tube with 1.5 mm Zirconium beads and the BeadBug 6 homogenizer from Benchmark Scientific, TEquipment Inc. (Long Branch, NJ, USA) Two highly correlated methods were employed to determine viral titers. The first method involved extracting total RNA from the homogenized tissues using the RNeasy Plus Mini kit (Qiagen Cat # 74136, Hilden, Germany). This RNA was then reverse transcribed with the iScript advanced cDNA kit (Bio-Rad Cat #1725036, Hercules, CA, USA). Subsequently, a SYBR Green Real-time PCR assay was conducted to quantify the copies of SARS-CoV-2 N gene RNA. The primers used were SARS-CoV-2 N F: 5′-ATGCTGCAATCGTGCTACAA-3′ and SARS-CoV-2 N R: 5′-GACTGCCGCCTCTGCTC-3′. All SYBR Green-based real-time PCR assays incorporated melt-curve analyses to ensure the detection of specific PCR products and avoid false positives. The second method utilized nLuc activity as a substitute for the plaque assay. Clarified tissue homogenates were serially diluted and used to infect Vero-E6 cell culture monolayers. After infection, the cells were washed with PBS and lysed using 1X Passive lysis buffer. The nLuc activity was then measured as previously described. Data analysis and visualization were performed using GraphPad Prism 8 v8.4.3.

### 2.14. mRNA Expression Analyses of Signature Inflammatory Cytokines and Lung Injury/Repair Genes

During necropsy, samples of brain and lung tissue were collected from mice. Approximately 20 mg of tissue was immersed in 500 µL of RLT lysis buffer, and RNA extraction was performed using the RNeasy Plus Mini kit (Qiagen Cat # 74136). The RNA was then converted to cDNA using the iScript advanced cDNA kit (Bio-Rad Cat #1725036). To quantify the mRNA levels of specific inflammatory cytokines and lung pathology markers, multiplex qPCR was conducted using the iQ Multiplex Powermix (Bio-Rad Cat # 1725848) and PrimePCR Probe Assay mouse primers for various *Gapdh, Il6, Ccl2, Cxcl10, Ifnɣ, Il1b, Krt8, Krt5, Adamts4, Itga5*. The reaction plate was analyzed using a CFX96 touch real-time PCR detection system, with all channels scanned. The PCR protocol consisted of an initial 2-min denaturation at 95 °C, followed by 40 cycles of 10 s at 95 °C and 45 s at 60 °C. A melting curve analysis confirmed the amplification of a single PCR product for each primer pair. The mRNA levels of *Il6*, *Ccl2*, *Cxcl10*, *Ifnɣ*, and *Il1b* in infected mice cDNA samples were normalized to *Gapdh* mRNA using the ΔCt method. The fold change was calculated using the 2^−ΔΔCt^ method, comparing infected mice to uninfected controls.

### 2.15. Disease Burden and Bliss Index Scores

Computation of disease burden (Figure S1G and Table 1) was carried out as reported previously [34]. These included measurements of viral loads [N mRNA levels, titers (nLuc activity)] and inflammatory responses [*Ccl2* and *Cxcl10* mRNA levels] in both brain and lung tissues. We also assessed lung damage (*Krt8* and *Adamts4* mRNA levels) and mortality for Figure S1G, bringing the total to 9 parameters. The disease index data shown in Table 1 was generated by using 7 parameters and included viral titers (nLuc activity)] and inflammatory responses [*Ccl2* and *Cxcl10* mRNA levels] in both cerebral and pulmonary tissues and mortality for WA1, Delta, and Beta SARS-CoV-2 infected mice. A total of 6 parameters were used for Omicron VOC-infected mice. Mortality was excluded as Omicron infection is not fatal in K18-hACE2 mice. For each metric, we standardized the data by assigning a value of 100 to the results from the control group (either untreated or given adjuvant). The overall disease impact was determined by adding the standardized values of each parameter (viral load, inflammation, lung damage, mortality). The sum was then divided by 6, 7, or 9, corresponding to the number of parameters evaluated, to normalize the data.

$$Total\;Disease\;burden=[\%\;reduction\;({Titre}_{lung}+{Titre}_{brain}+{Ccl2\;mRNA}_{lung}+{Ccl2mRNA}_{brain}+\;{Cxcl10\;mRNA}_{lung}+{Cxcl10\;mRNA}_{brain}+K{rt8\;mRNA}_{lung}+Adamts4\;{\;mRNA}_{lung})+\%\;Mortality]/9.$$

To determine if the combinatorial vaccine regimen provided benefits over the intranasal or intramuscular vaccination regimen, we utilized Bliss index scores (Figure S1G) based on the overall disease burden under each condition. Bliss index scores were calculated as conducted previously [34,41] using the formula below:

$$\begin{array}{c} Bliss\;index_{Regimen(i.n.+i.m.)}\\ \;\;\;\;\;\;={\%\;Inhibition\;in\;Disease\;burden}_{Regimen(i.n.+i.m.)}\\ \;\;\;\;\;\;-100(1-(1\\ \;\;\;\;\;\;-\frac{{\%\;Inhibition\;in\;Disease\;burden}_{Regimen(i.n.)}}{100})(1\\ \;\;\;\;\;\;-\frac{{\%\;Inhibition\;in\;Disease\;burden}_{Regimen\;(i.m.)}}{100})) \end{array}$$

The Bliss index shown in Figure S1G was used to categorize interactions as follows: scores below −10 indicated antagonism, scores ranging from −10 to +10 signified an additive effect, and scores exceeding 10 denoted synergy.

### 2.16. Cryo-Electron Tomography of SARS-CoV-2 VLPs

Concentrated SARS-CoV-2 SMEN particles mixed with fiducial markers were placed onto glow-discharged holey carbon grids. Grids were blotted with filter paper and plunge-frozen in liquid ethane by a homemade gravity-driven plunger. Tomographic tilt series were collected between −51° and +51° with a 3° step using SerialEM [42] in a dose-symmetric scheme [43] on a cryo-TEM (Titan Krios, Thermo Fisher Scientific, Waltham, MA, USA). Images were captured at a defocus of −0.5 um with an energy-filtered Gatan K3 direct detector and a Volta Phase Plate (VPP) at a pixel size of 1.346 Å. The accumulative dose was ~120 e per Å2 for a single tilt series, with 8 frames saved for each tilt angle. Tomograms were reconstructed and visualized with IMOD [44] after motion correction using Motioncorr2 [42,43,44,45].

### 2.17. Quantification and Statistical Analysis

Data were analyzed and graphed using the software program GraphPad Prism 8 v8.4.3 (La Jolla, CA, USA). Pairwise comparisons were assessed for statistical significance using the non-parametric Mann-Whitney test with a two-tailed approach. To determine the statistical significance of the survival curves, the log-rank (Mantel-Cox) test was employed. A two-way ANOVA was conducted to analyze grouped data, followed by Tukey’s multiple comparison tests. Statistical significance was established at *p* < 0.05. *p* values are indicated as: *, *p* < 0.05; **, *p* < 0.01; ***, *p* < 0.001; ****, *p* < 0.0001.

## 3. Results

### 3.1. Systemic and Mucosal Vaccination of the SARS-CoV-2 VLPs (SMEN) Vaccine Provides Enhanced Protection in K18-hACE2 Mice against a Lethal Virus Challenge

**Figure 1:**
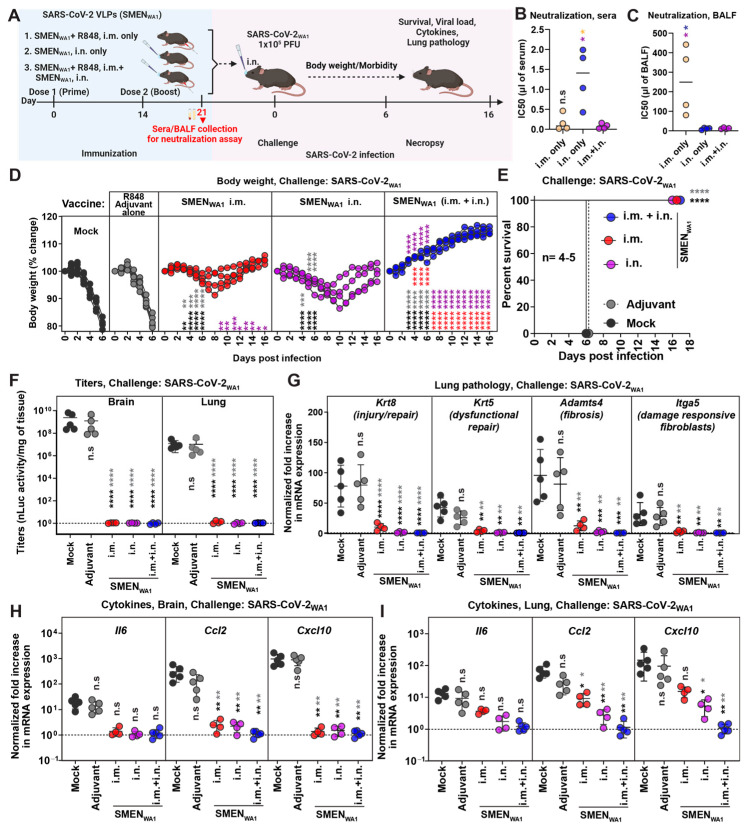
Enhanced Protection Against Lethal SARS-CoV-2 Challenge in K18-hACE2 Mice via Systemic and Mucosal SMEN Vaccine Administration (**A**) A scheme showing experimental design for comparing protective efficacy of SAR-CoV-2 VLP (SMEN_WA1_) vaccine using various routes. A total of 250 µg of SMEN vaccine was used to immunize K18-hACE2 mice as depicted intramuscularly (i.m.) or i.n. using the prime-boost strategy. A total of 30 μg of vaccigrade TLR7/8 agonist R848 was used as an adjuvant for the i.m. route. Adjuvant and untreated mice served as control groups. A total of 21 days after immunization, sera/ bronchioalveolar lavage fluids (BALF) were harvested (before challenge), or mice challenged i.n. with 1 × 10^5^ PFU of SARS-CoV-2_WA1_ and indicated parameters were analyzed to assess protection. (**B**,**C**) Neutralizing titers (IC50) in the sera and BALF from indicated groups of vaccinated as in (**A**). (**D**) The graph illustrates the changes in body weight over time for K18-hACE2 mice following infection. Each line represents an individual animal, with the initial weight set at 100%. (**E**) The plot displays Kaplan-Meier survival curves for the experiment described in (**A**). (**F**) The graph shows the viral load measurements in various organs at the time of necropsy, expressed as nLuc activity per milligram of tissue, using Vero-E6 cells as targets. (**G**) Fold change in mRNA expression of indicated lung pathology markers, *Krt8*, *Krt5*, *Adamts4,* and *Itga5* in the lung tissue after necropsy. (**H**,**I**) Changes in specific cytokine mRNA expression levels were measured in mouse brain and lung tissues following specified treatment protocols. These measurements were taken either at the time of death or at 16 dpi for surviving mice. The data were standardized against *Gapdh* mRNA from the same sample and from uninfected mice after necropsy in the experiments depicted in (**G**–**I**). Statistical analysis for data in (**B**,**C**) employed the nonparametric Mann-Whitney test. For grouped data in (**D**) and (**F**–**I**), a two-way ANOVA was conducted, followed by Tukey’s multiple comparison tests to assess significance. Statistical significance for the group comparisons to mock is shown in black, with adjuvant shown in light dark, with SMEN_WA1_ i.m. shown in red, and SMEN_WA1_ i.n. shown in magenta. *, *p* < 0.05; **, *p* < 0.01; ***, *p* < 0.001; ****, *p* < 0.0001; ns, not significant; Mean values ± SD are depicted.

We sought to investigate the efficacy of the SMEN vaccine in providing protection against lethal challenges in the highly susceptible K18-hACE2 mouse model of infection. Towards this end, we first evaluated systemic [intramuscular (i.m.; adjuvanted)], mucosal [intranasal (i.n.; unadjuvanted)] or combined (i.m. + i.n.) vaccination regimen to determine if mucosal immunity contributes to protection (Figure 1A). We used the TLR7/8 agonist R848 (Resiquimod; vaccigrade) as an adjuvant for intramuscular administrations. For the intranasal route, SMEN particles were administered without any adjuvant to avoid adjuvant-triggered inflammatory responses in the lung. We produced ancestral SARS-CoV-2 VLPs (SMEN_WA1_) by co-transfecting individual plasmids encoding the SARS-CoV-2 spike (S_WA1_), membrane (M), envelope (E), and nucleocapsid (N) proteins followed by purification via high-speed sedimentation through a sucrose cushion (Figure S1A). Analyses of SMEN particles by western blot confirmed the packaging of the four viral proteins, while cryo-electron tomography (cryoET) illustrated the presence of prefusion S protein on the particles (Figure S1B,C). We followed a prime-boost strategy of vaccination and analyzed neutralizing antibody (nAb) titers in the sera and bronchioalveolar lavage fluid (BALF) (Figure 1A). Our analyses revealed distinct patterns of nAb titers. While intramuscular vaccination elicited strong nAb titers in sera but significantly reduced levels in BALF, intranasal vaccination induced robust nAb titers in BALF but diminished titers in the sera. Notably, the combinatorial administration resulted in robust and higher nAb titers in both the sera and BALF of mice (Figure 1B,C, Supplementary Figure S1D,E, Table 1).

To assess the potential implications of divergent nAbs titers in the two compartments on protective immunity, both control [mock, no treatment, and adjuvant alone] and vaccinated groups of mice were challenged intranasally with a lethal dose of SARS-CoV-2_WA1_ (Figure 1A). Our analyses revealed that all the mice in the control group exhibited severe morbidity and demonstrated up to 20% loss from their starting body weight by day 6 and succumbed to SARS-CoV-2-induced mortality. Notably, the course of infection in mice treated with adjuvant alone was unaltered and similar to untreated mice, eliminating the concerns of innate immune memory from our analyses [46,47]. In comparison, cohorts of mice immunized either via systemic or mucosal routes experienced only a transient body weight loss (~5–10%) between days 6 and 10 post-challenge and eventually recovered from infection and survived. Mice in the combined administration group did not experience any weight loss, indicating near-complete protection from infection (Figure 1D,E). These data were corroborated by our assessment of viral loads (Titers and N mRNA expression) in the brain and lung tissues, which revealed significantly low or undetectable viral RNA in either tissue for all mice in the SMEN-immunized groups (Figure 1F and Figure S1F). To assess lung pathology, we utilized specific markers for various parameters that were identified in recent transcriptomics studies as substitutes [34,48,49,50,51,52,53]. Specifically, we used mRNA expression of *Krt8* (indicative of injury/repair), *Krt5* (related to dysfunctional repair), *Adamts4* (associated with fibrosis), and *Itga5* (indicative of damage-responsive fibroblasts). Our data revealed that mice immunized with SMEN, irrespective of the administration route, exhibited significantly lower mRNA expression of all the analyzed markers compared to those in the control group at the time of necropsy (Figure 1G). Of the vaccinated groups, only mice immunized intramuscularly exhibited 5–20-fold higher mRNA expression levels of *Krt8* and *Adamts4* compared to uninfected mice, suggesting relatively lower protection in the lung conferred by systemic vaccination compared to the mucosal route. Moreover, mRNA expression of inflammatory cytokines in the brain was significantly reduced in all three groups of vaccinated mice compared to those in the control group, suggesting virologic control in the brain (Figure 1H,I). Although the overall mRNA expression of inflammatory cytokines in the lung was significantly reduced compared to the control groups, we observed a graded reduction in the vaccinated groups, with the combined regimen demonstrating the most substantial decrease (similar to uninfected mice), followed by the mucosal route (9-fold reduction) and then the systemic route (2-fold reduction) (Figure 1H,I). We computed the total disease burden for mice under each regimen using eight parameters (titers in brain and lung; injury markers (*Krt8*, *Adamts4*); cytokine expression (*Ccl2, Cxcl10*) in lung and brain) as previously described [34]. Our evaluation revealed a significant overall reduction in total disease burden between the control and vaccinated groups, with the combined regimen displaying the highest reduction among all the groups (Table 1). Additionally, we calculated Bliss index scores to determine whether the combined regimen offered any advantage over the individual regimens alone (Figure S1G). Our analysis revealed a Bliss index score of −0.4 (Bliss index score range: −10 to 10 denotes additivity), suggesting that the combined regimen exhibited an additive efficacy in vivo (Figure S1G).

Thus, the data underscored the effectiveness of mucosal versus systemic vaccine regimens in protecting the lung from SARS-CoV-2-induced pathology. Our data also demonstrated the superior overall efficacy of the combined regimen in reducing SARS-CoV-2-induced disease burden. Consequently, we adopted the combined vaccination regimen for the remainder of our study.

### 3.2. Antibodies Play a Major Role in SMEN Vaccine-Mediated Protection

**Figure 2:**
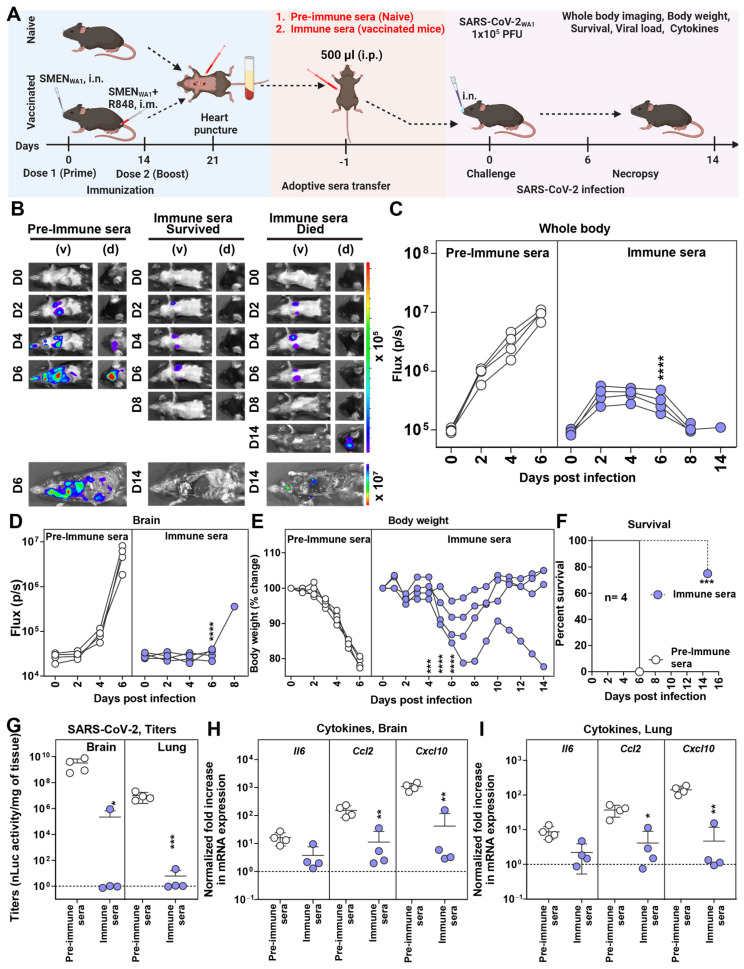
SMEN Vaccine-Elicited Antibodies Can Protect Naïve Mice from SARS-CoV-2-Induced Mortality (**A**) Experimental design for testing the contribution of immune sera to conferring protection. Pre-immune sera (500 µL) or sera from vaccinated mice (immune sera) were passively transferred into naïve K18-hACE2 mice one day before challenge with SARS-CoV-2_WA1-nLuc_ (i.n., 1 × 10^5^ PFU). (**B**) The BLI images show nLuc signals in mice infected with SARS-CoV-2_WA1-nLuc_ in ventral (v) and dorsal (d) view. (**C**,**D**) Non-invasive measurement of nLuc signal as flux (photons/sec) over time. (**E**) The graph illustrates the changes in body weight over time for K18-hACE2 mice following infection. Each line represents an individual animal, with the initial weight set at 100%. (**F**) The plot displays Kaplan-Meier survival curves for the experiment described in (**A**). (**G**) The graph shows the viral load measurements in various organs at the time of necropsy, expressed as nLuc activity per milligram of tissue, using Vero-E6 cells as targets. (**H**,**I**) Changes in specific cytokine mRNA expression levels were measured in mouse brain and lung tissues following specified treatment protocols. These measurements were taken either at the time of death or at 16 dpi for surviving mice. The data were standardized against *Gapdh* mRNA expression from the same sample and from uninfected mice after necropsy in the experiments depicted in (**G**,**I**). A statistical analysis was conducted for grouped data in (C–E) and (G–I), and a two-way ANOVA was conducted, followed by Tukey’s multiple comparison tests to assess significance. Statistical significance for the group comparisons to pre-immune sera is shown in black. *, *p* < 0.05; **, *p* < 0.01; ***, *p* < 0.001; ****, *p* < 0.0001; ns, not significant; Mean values ± SD are depicted.

The combined regimen of SMEN vaccine delivery elicited potent nAbs in both BALF and sera (Figure 1B,C). To ascertain if antibodies contributed to vaccine-mediated protection, we investigated whether passive transfer of sera from vaccinated mice can confer protection in naïve mice against SARS-CoV-2-induced mortality. To gain deeper insights into the impact of sera on virus replication and dissemination, we used our bioluminescence imaging (BLI)-guided platform, which enables real-time longitudinal monitoring of virus replication using nanoLuc luciferase (nLuc)-expressing reporter SARS-CoV-2_WA1-nLuc_ [34,37,38]. Pre-immune sera or sera from vaccinated mice (immune sera) were collected and adoptively transferred (i.p.) to naïve K18-hACE2 mice one day before the lethal challenge with SARS-CoV-2_WA1-nLuc_ (Figure 2A)_._ BLI and nLuc flux quantification showed that the pre-immune sera-treated groups experienced uncontrolled virus replication in the lungs, which spread to the brain, leading to more than 20% body weight loss and 100% mortality. (Figure 2B–F). In contrast, 75% of mice treated with immune sera exhibited reduced virus replication from 2 to 6 dpi in the lung, followed by its clearance at 8 dpi (Figure 2B,C and Figure S2). Although one mouse in the group receiving immune sera exhibited a below-detection nLuc signal in the lung at 8 dpi, it lost body weight after a brief recovery period from 8 to 10 dpi. BLI revealed that the virus had spread to the brain, resulting in its death (Figure 2B–F and Figure S2). Viral load and inflammatory cytokine mRNA analyses in the target tissues corroborated our BLI analysis and revealed significant control of virus replication and inflammation in the mice receiving immune sera (Figure 2G–I and Figure S2C). Therefore, our analyses indicate that the humoral immune response elicited by the SMEN vaccine plays a major role in curbing virus replication and establishing immune control.

### 3.3. CD8^+^ and CD4^+^ T Cells Elicited by SMEN Vaccine Play a Minor but Distinct Role in Immunity by Contributing to Diminishing Virus Spread and Reducing Inflammation

**Figure 3:**
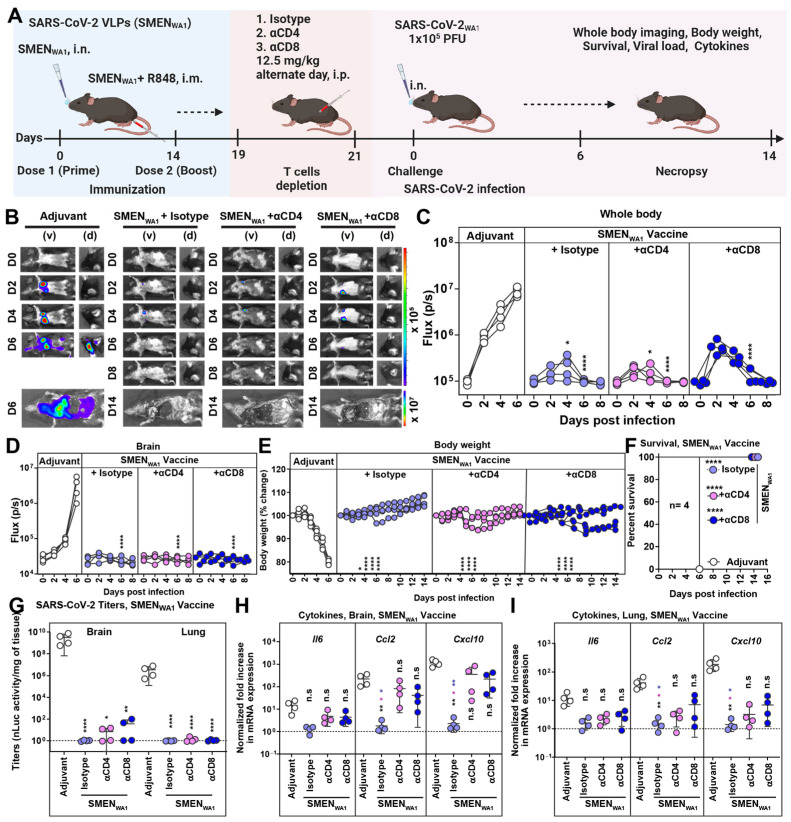
Minor Yet Distinct Role of CD8^+^ and CD4^+^ T Cells in SMEN_WA1_ Vaccine-mediated Protection. (**A**) Experimental design to test the contribution of CD4^+^ and CD8^+^ T cells in SMEN-vaccine meditated protection. K18-hACE2 mice were immunized as shown using the combined regimen with SMEN_WA1_. Adjuvant-treated mice served as control. αCD4 and αCD8alpha mAbs (12.5 mg/kg body weight, i.p.) were used to deplete CD4^+^ and CD8^+^ T cells every 48-h starting at 19 days post-vaccination. Isotype mAb treated cohorts served as controls (Isotype). The mice (n = 4 each group) were challenged with 1 × 10^5^ PFU of SARS-CoV-2_WA1-nLuc_ and followed by non-invasive BLI every 2 days from the start of infection till 8 dpi. (**B**) BLI images show nLuc signals in mice infected with SARS-CoV-2_WA1-nLuc_ in ventral (v) and dorsal (d) view. (**C**,**D**) Non-invasive measurement of nLuc signal as flux (photons/sec) over time (**E**) The graph illustrates the changes in body weight over time for K18-hACE2 mice following infection. Each line represents an individual animal, with the initial weight set at 100%. (**F**) The plot displays Kaplan-Meier survival curves for the experiment described in (**A**). (**G**) The graph shows the viral load measurements in various organs at the time of necropsy, expressed as nLuc activity per milligram of tissue, using Vero-E6 cells as targets. (**H**,**I**) Changes in specific cytokine mRNA expression were measured in mouse brain and lung tissues following specified treatment protocols. These measurements were taken either at the time of death or at 16 dpi for surviving mice. The data were standardized against *Gapdh* mRNA from the same sample and from uninfected mice after necropsy in the experiments depicted in (**G**–**I**). A statistical analysis was conducted for grouped data in (**C**–**E**) and (**G**–**I**), and a two-way ANOVA was conducted, followed by Tukey’s multiple comparison tests to assess significance. Statistical significance for the group’s comparisons to adjuvant are shown in black, with the CD4^+^ T-cells depleted group shown as magenta and with the CD8^+^ T-cells depleted group shown as blue. *, *p* < 0.05; **, *p* < 0.01; ****, *p* < 0.0001; ns, not significant; Mean values ± SD are depicted.

Next, we evaluated the involvement of T cells in contributing to SMEN vaccine-mediated immunity (Figure 3A). Towards this end, we immunodepleted subtypes of T cells by treating vaccinated mice with CD4^+^ or CD8^+^ T cell-depleting antibodies. Mice treated with isotype antibodies served as control. After confirming the specific depletion of CD4^+^ or CD8^+^ T cells in the blood by flow cytometry (Figure S3A,B), we challenged the groups of mice with a lethal dose of SARS-CoV-2_WA1-nLuc_. Longitudinal imaging and quantification of nLuc signals revealed expansion of virus infection in the lung after its initial appearance at 2 dpi and dissemination to the brain by 4–6 dpi in the adjuvant-treated mice (Figure 3B–D). In comparison, the isotype Ab-treated vaccinated mice demonstrated significantly lower nLuc signals in the lung that were quickly controlled without virus dissemination to the brain. Non-invasive imaging indicated that virologic control remained comparable after T cell depletion, implying a minimal role of T cells in SMEN-mediated protection. However, the depletion of CD8^+^ T cells resulted in detectable and increased nLuc signals in the lungs of the vaccinated groups, suggesting a greater contribution of CD8^+^ T cells compared to CD4^+^ T cells in protection (Figure 3B–D). Body weight analyses and survival data confirmed the minimal role of T cells, as all mice in the vaccinated and T cell-depleted group survived lethal challenges (Figure 3E,F). Imaging of isolated organs post-necropsy, which allows for improved sensitivity, revealed sustained virologic control in the lungs and was confirmed by quantification of N mRNA expression despite T cell depletion (Figure S3C–E). However, significantly lower yet detectable nLuc signals were observed in the brain of T cell-depleted mice, in contrast to mice in the isotype-treated groups that demonstrated complete virologic control (Figure S3C). This was also evident in the detectable viral loads and expression of viral N mRNA in the brain following T cell depletion (Figure 3G and Figure S3E). In addition, mRNA expression of inflammatory cytokines *Ccl2* and *Cxcl10* remained significantly higher than that of isotype-treated vaccine mice in both the lung and brain (Figure 3H,I). Thus, considering the lesser impact on SARS-CoV-2-induced mortality, our data suggest that SMEN vaccine-elicited T cells play a minor yet distinct role in immunity by helping to reduce the spread of the virus to the brain and mitigating inflammatory responses in infected tissues.

### 3.4. Cross-Protective Efficacy of SMEN_WA1_ Vaccine Is Reduced against Delta and Ineffective against Beta VOC

**Figure 4:**
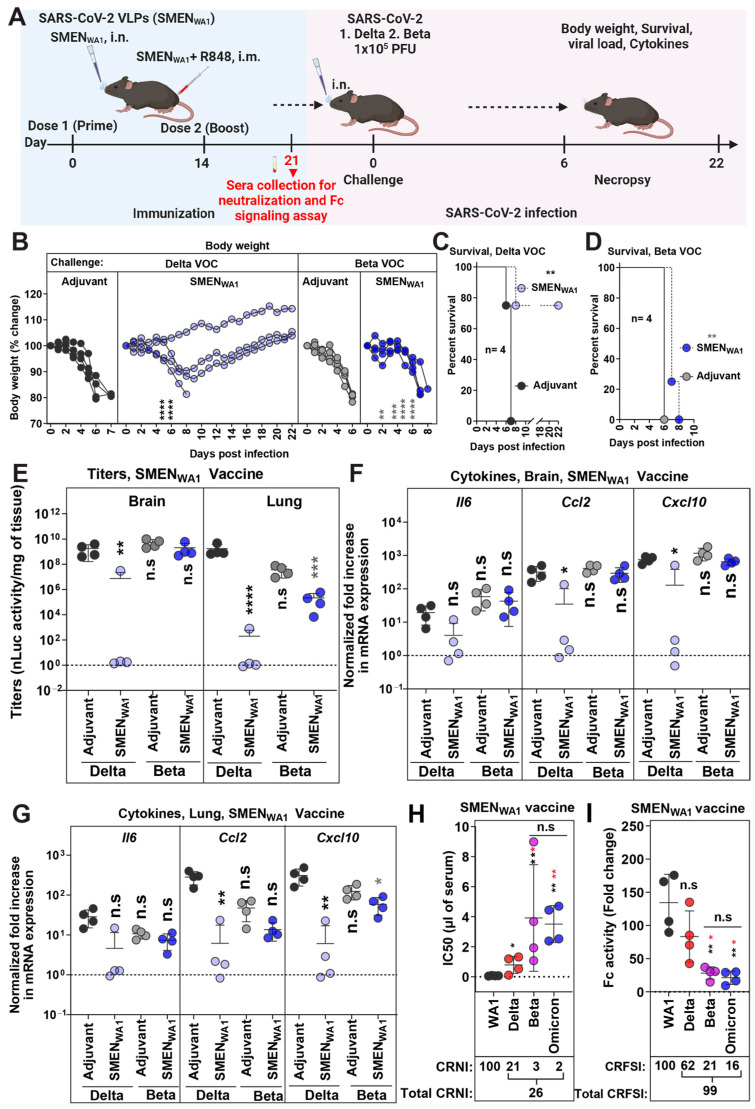
SMEN_WA1_ Vaccine is Effective Against Delta and Provides Limited Cross-protection Against Beta VOC. (**A**) Experimental design for testing cross-protection efficacy of SMEN_WA1_vaccine against Delta and Beta VOCs. The mice were challenged with 1 × 10^5^ PFU of Delta and Beta SARS-CoV-2 nLuc to SMEN_WA1_ vaccinated mice. (**B**) The graph illustrates the changes in body weight over time for K18-hACE2 mice following infection. Each line represents an individual animal, with the initial weight set at 100%. (**C**,**D**) The plot displays Kaplan-Meier survival curves for the experiment described in (**A**). (**E**) The graph shows the viral load measurements in various organs at the time of necropsy, expressed as nLuc activity per milligram of tissue, using Vero-E6 cells as targets. (**F**–**G**) Changes in specific cytokine mRNA expression levels were measured in mouse brain and lung tissues following specified treatment protocols. These measurements were taken either at the time of death or at 16 dpi for surviving mice. The data were standardized against *Gapdh* mRNA from the same sample and from uninfected mice after necropsy in the experiments depicted in (**E**–**G**). (**H**) Plot showing live virus neutralizing IC50 (µL of serum) values in the sera from SMEN_WA1_ vaccinated mice before challenge, (see scheme in (**A**), n = 4; each dot represents one mouse; 21 days post-vaccination) against WA1, Delta, and Beta, Omicron variants. Cross-reactive neutralization index (CRNI) values shown below the plot were calculated (see Section 2) by setting the homologous WA1 strain to 100. (**E**) Fold change in FcγR signaling measured using Jurkat NFAT-luciferase (JNL) cells expressing mFcγRIV, co-cultured with Vero E6 cells infected with the indicated SARS-CoV-2 variants and sera (IC50 equivalent) from SMEN_WA1_-vaccinated mice before challenge (21 days post-vaccination). Data were normalized to luciferase activity measured in the absence of sera. To estimate the cross-reactive Fc-signaling index (CRFSI), the Fc-signaling activity measured using Vero E6 cells infected with the homologous WA1 strain was set to 100. A statistical analysis was conducted for grouped data in (**B**) and (**E**–**G**), and a two-way ANOVA was conducted, followed by Tukey’s multiple comparison tests to assess significance. Statistical analysis for data in (**H**,**I**) employed the nonparametric Mann-Whitney test. Statistical significance for the group comparisons in (**B**–**G**) to adjuvant-treated mice with Delta VOC-infected mice are shown in black, and adjuvant-treated mice with Beta VOC-infected mice is shown in grey. Statistical significance for the group comparisons in (**H**–**I**) to WA1 are shown in black, and Delta VOC infected mice are shown in red. *, *p* < 0.05; **, *p* < 0.01; ***, *p* < 0.001; ****, *p* < 0.0001; ns, not significant; Mean values ± SD are depicted.

The development of hybrid immunity through infection and/or vaccination complicates the assessment of cross-protective efficacy solely attributable to vaccine- or variant-specific spike protein-generated immune responses. Lethal mouse models of SARS-CoV-2 infection, like K18-hACE2 mice, fill this gap and allow in vivo evaluation for cross-protective immunity in a naïve background. For evaluating cross-protective efficacies, we generated SMEN-VLPs carrying spike protein from Delta, Beta, and Omicron VOCs in addition to the above-described SMEN_WA1_ and validated them using western blot analyses (Figure S4A). As a first step, we evaluated the cross-protective efficacy of the SMEN_WA1_ vaccine against Beta and Delta VOC, which carried distinctive nAb-resistant mutations in S and arose during the pandemic in separate geographic areas [54]. We challenged adjuvant-treated or SMEN_WA1_-vaccinated K18-hACE2 mice intranasally with a lethal dose of Beta or Delta VOC (Figure 4A). Evaluation of physical parameters revealed that adjuvant-treated mice exhibited steady body weight loss and succumbed to infection upon Delta or Beta VOC challenge (Figure 4B). However, 75% of vaccinated mice recovered and survived the lethal Delta VOC challenge despite the 10–15% initial loss in body weight from 2 to 8 dpi. Accordingly, vaccination with SMEN_WA1_ protected 75% of the mice against Delta VOC-induced mortality (Figure 4C,D). The reduction of viral loads (N mRNA expression and titers in the lung and brain of surviving Delta VOC challenged mice was significantly low and near the limit of detection, indicating virus clearance (Figure 4E and Figure S4B). Moreover, inflammatory cytokine expression (*Ccl2*, *Cxcl10*) in the lung and brain was also significantly reduced (Figure 4F,G). In contrast, Beta VOC was highly resistant to SMEN_WA1_-elicited immunity, causing a steady loss in body weight and resulting in 100% mortality among the vaccinated mice (Figure 4B–D). Only one mouse experienced a 2-day delay in death. In addition, the vaccinated mice showed 4 logs of higher Beta VOC viral loads in the lung and the brain than uninfected controls. This was despite significantly lower viral loads (1–2 logs) than adjuvant-treated groups, indicating impaired virologic control (Figure 4E and Figure S4B). In addition, inflammatory cytokine mRNA expression was also 1–3 logs higher than in uninfected mice despite a significant reduction compared to adjuvant-treated mice (Figure 4F,G). As humoral immune responses played a major role in protection (Figure 2), we also analyzed Delta, Beta, and Omicron (BA.1) (most divergent in our study) VOC cross-reactive activities in the sera of SMEN_WA1_ vaccinated mice before the challenge. Western blot analyses showed that sera from SMEN_WA1_-vaccinated mice detected all the proteins in SMEN particles, including spike proteins from heterologous variants (Figure S4C). These data demonstrated successful immunization and generation of expected immune response. We then interrogated both the neutralizing and Fc-signaling index as several previous studies have indicated that both neutralizing and Fc-effector functions of antibodies contribute to protection against SARS-CoV-2 (Figure 4H,I and Figure S4D, Table 1). For our comparative analyses of the cross-reactive index, the neutralizing and Fc signaling activity against the homologous WA1 strain was set to 100. The data indicated that while cross-neutralization activity against all VOCs was overall significantly diminished (cross-reactive neutralizing index, CRNI = 26), the loss in cross-neutralizing activity against Beta and Omicron VOCs was severe (CRNI= 2 and 3, respectively) compared to Delta VOC (CRNI = 21) (Figure 4H, Table 1). A similar decline in the overall cross-reactive Fc-signaling index (CRFSI = 99) was observed for Fc-signaling activity in the SMEN_WA1_-elicited sera. The sera demonstrated better activity against the Delta VOC (CRFSI = 62 vs. 100 for WA1) compared to the Beta and Omicron VOCs, where the cross-reactive indices were significantly lower (CRFSI = 21 and 16, respectively (Figure 4I). These data explained the contrasting outcome and higher protection seen against Delta VOC compared to Beta VOC, resulting in lower disease burden (9.5 vs. 48.8) (Table 1). Together, our data show that the SMEN_WA1_ vaccine has a reduced capacity to provide immunity against Beta and Omicron variants and explains the superior resistance of Beta VOC for causing breakthrough infections, as noted in our in vivo experiments in mice.

### 3.5. SMEN_Omicron_ Vaccine Provides Diminished Cross-Protection against Previous Variants of SARS-CoV-2

**Figure 5:**
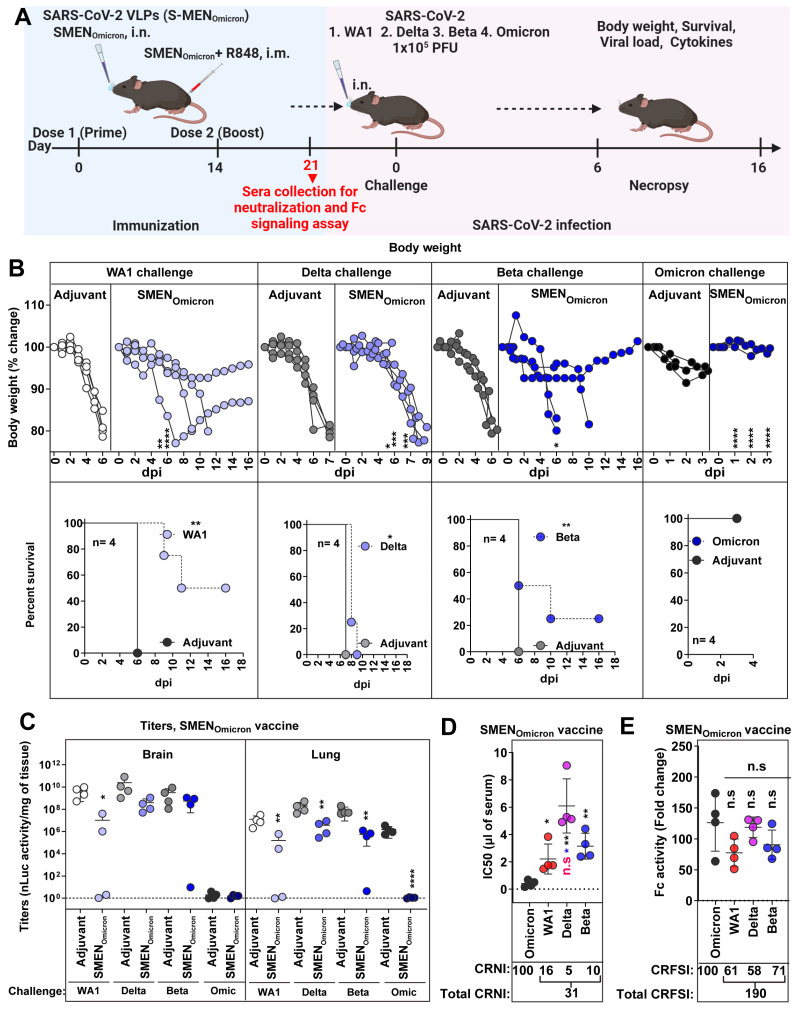
SMEN_Omicron_ Provides Weak Cross-protection Against Heterologous VOCs. (**A**) Experimental design for testing cross-protection efficacy of SMEN_Omicron_ vaccine against WA1, Delta, Beta, and Omicron VOCs. Indicated groups of mice (control and vaccinated) were challenged with 1 × 10^5^ PFU of SARS-CoV-2 nLuc variants (WA1, Delta, Beta, and Omicron) (**B**) The graph illustrates the changes in body weight (top panel) over time for K18-hACE2 mice following infection. Each line represents an individual animal, with the initial weight set at 100%, and the plot displays Kaplan-Meier survival curves for the experiment described in (**A**) is shown in (bottom panel). (**C**) The graph shows the viral load measurements in various organs at the time of necropsy, expressed as nLuc activity per milligram of tissue, using Vero-E6 cells as targets. (**D**) Plot showing neutralizing IC50 (µL of serum) values in the sera from SMEN_Omicron_ vaccinated mice harvested before challenge (see scheme in (**A**), n = 4, each dot represents one mouse; 21 days post-vaccination) against Omicron, WA1, Delta and Beta variants. Cross-reactive neutralization index (CRNI) values shown below the plot were calculated (see Section 2) by normalizing the homologous Omicron variant to 100. (**E**) Fold change in FcγR signaling measured using Jurkat NFAT-luciferase (JNL) cells expressing mFcγRIV, co-cultured with Vero E6 cells infected with the indicated SARS-CoV-2 variants and sera (IC50 equivalent) from SMEN_Omicron_-vaccinated mice harvested before challenge at 21 days post-vaccination. Data were normalized to luciferase activity measured in the absence of sera. To estimate the cross-reactive Fc-signaling index (CRFSI), the Fc-signaling activity measured using Vero E6 cells infected with the homologous Omicron strain was set to 100. A statistical analysis of grouped data in (**B**,**C**) using a two-way ANOVA was conducted, followed by Tukey’s multiple comparison tests to assess significance. Statistical analysis for data in (**D**,**E**) employed the nonparametric Mann-Whitney test. Statistical significance for the group comparisons to adjuvant with each cohort is shown in black. Statistical significance for the group comparisons in (**D**,**E**) to Omicron is shown in black, WA1 is shown in red, and beta is shown in blue. *, *p* < 0.05; **, *p* < 0.01; ****, *p* < 0.0001; ***, *p* < 0.001; ns, not significant; Mean values ± SD are depicted.

The Omicron variants have a constellation of mutations in S (37 mutations in Omicron BA.1 compared to WA1) [55] that allows evasion of vaccine/infection-induced immunity from previous variants. Hence, we explored if the SMEN vaccine with the more divergent Omicron spike can elicit broader immunity and demonstrate better protection. Towards this end, we challenged adjuvant-treated and SMEN_Omicron_ vaccinated K18-hACE2 mice (combined regimen) with ancestral (WA1), Delta, Beta, and Omicron (BA.1) variants. The Omicron VOC does not produce a lethal infection in the K18-hACE2 mouse model, and the mice recover in 3–4 days after exhibiting a brief reduction in body weight that peaks at 2 dpi [34]. We, therefore, terminated the Omicron VOC-challenged cohorts of mice at 3 dpi (Figure 5A). Our data revealed that immunity elicited by the SMEN_Omicron_ vaccine was effective against Omicron VOC as immunization prevented body weight loss and significantly reduced viral loads in the lungs compared to adjuvant-treated mice (Figure 5B,C). Surprisingly, the SMEN_Omicron_ vaccine did not offer 100% protection against the three strains tested. It provided limited protection, with survival rates of 50%, 0%, and 25% with delayed death against lethal challenges with the ancestral, Delta, and Beta variants, respectively (Figure 5B). The viral loads mirrored the survival data, showing diminished virologic control for all three strains tested, except for the homologous Omicron VOC, where the vaccine demonstrated the highest efficacy (Figure 5C and S5A). Analysis of pro-inflammatory cytokines corroborated these observations and revealed that the SMEN_Omicron_ vaccine reduced mRNA expression of *Ccl2* (1.7–6-fold in the brain and 2.5–5.3-fold in the lung) and *Cxcl10* (1.8–10.1-fold in brain and 1.5–7.4-fold in the lung) when challenged with heterologous SARS-CoV-2 strains. However, the SMEN_Omicron_ vaccine was significantly more effective in reducing inflammation (22–26-fold reduction in the brain; 6.5–14.3-fold in the lung) when challenged with homologous strain (Figure S5B,C). Western blot analyses confirmed that sera from SMEN_Omicron_-vaccinated mice had reactivity against the proteins in SMEN particles, including spike proteins from heterologous variants, demonstrating successful immunization and elicitation of the expected immune response (Figure S5D). To understand the limited protection offered by the vaccine, we analyzed the sera from SMEN_Omicron_-vaccinated mice before the challenge for cross-reactive neutralizing and Fc-signaling activity (Figure 5D,E, Table 1). Indeed, the SMEN_Omicron_ vaccine exhibited a low total CRNI of 31. The CRNI was lowest against the Delta VOC (5), followed by Beta (10) and WA1 (16), mirroring the level of protection and overall disease burden observed in the survival analyses (Figure 5D, Table 1). Evaluation of SMEN_Omicron_-elicited sera for cross-reactive Fc-signaling index revealed broad activity against the three strains that totaled 190 (Figure 5E). The data imply that although the antibodies elicited by the SMEN_Omicron_ vaccine do not exhibit high cross-neutralizing activity against previous variants, which is a crucial first line of defense, they were able to bind S on infected cells and were capable of eliciting Fc receptor signaling. Overall, our data indicate that antibodies elicited by the Omicron Spike had a reduced ability to neutralize previous VOCs (Table 1) owing to its extensive divergence, making the SMEN_Omicron_ vaccine less effective against these earlier variants in mice.

### 3.6. SMEN_Beta_ Vaccine Provides Broad Cross-Protection against Heterologous Variants of SARS-CoV-2

**Figure 6:**
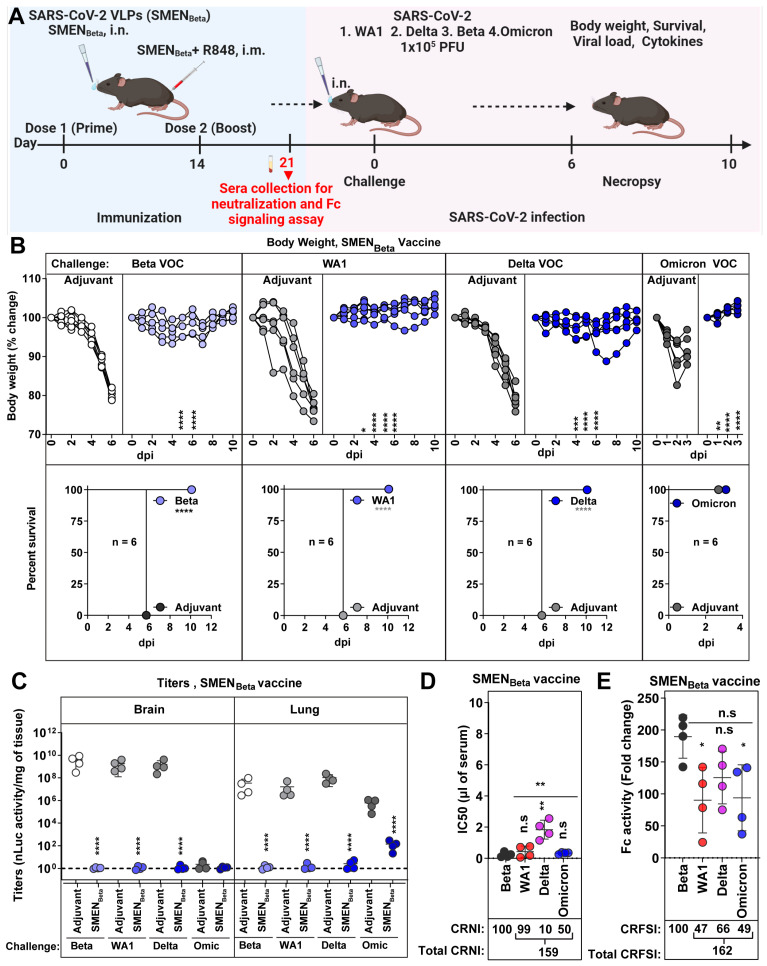
SMEN_Beta_ Vaccination Confers Cross-protection Against Ancestral Strain and Heterologous VOCs. (**A**) Experimental design for testing protection of SMEN_Beta_ vaccine against nanoluc expressing Beta, WA1, Delta, and Omicron reporter VOCs. SMEN_Beta_ vaccinated K18-hACE2 mice (combined regimen; n = 4) were challenged intranasally with 1 × 10^5^ PFU of indicated SARS-CoV-2 variants and subjected to indicated multiparametric analyses to determine efficacy (**B**) The graph illustrates the changes in body weight (top panel) over time for K18-hACE2 mice following infection. Each line represents an individual animal, with the initial weight set at 100%, and the plot displays Kaplan-Meier survival curves for the experiment described in (**A**) is shown in (bottom panel). (**C**) The graph shows the viral load measurements in various organs at the time of necropsy, expressed as nLuc activity per milligram of tissue, using Vero-E6 cells as targets. (**D**) Plot showing live virus neutralizing IC50 (µL of serum) values in the sera from SMEN_Beta_ vaccinated mice harvested before challenge at 21 days after immunization (see scheme in (**A**) and Table 1, n = 4) against Beta, WA1, Delta, and Omicron variants. Cross-reactive neutralization index (CRNI) values shown below the plot were calculated (see Section 2) by normalizing the mean IC50 values in the sera harvested before the challenge at 21 days after immunization for the homologous Beta variant to 100. (**E**) Fold change in live virus FcγR signaling assay measured using Jurkat NFAT-luciferase (JNL) cells expressing mFcγRIV, co-cultured with Vero E6 cells infected with the indicated SARS-CoV-2 variants and sera (IC50 equivalent) from SMEN_Beta_-vaccinated mice harvested before challenge at 21 days post-immunization. Data were normalized to luciferase activity measured in the absence of sera. To estimate the cross-reactive Fc-signaling index (CRFSI), the Fc-signaling activity measured using Vero E6 cells infected with the homologous Beta strain was set to 100. A statistical analysis of grouped data in (**B**,**C**) using a two-way ANOVA was conducted, followed by Tukey’s multiple comparison tests to assess significance. Statistical analysis for data in (**D**,**E**) employed the nonparametric Mann-Whitney test. Statistical significance for the group comparisons to adjuvant with each cohort is shown in black. Statistical significance for the group comparisons in (**D**,**E**) to beta are shown in black. *, *p* < 0.05; **, *p* < 0.01; ****, *p* < 0.0001; ***, *p* < 0.001; ns, not significant; Mean values ± SD are depicted.

The Beta VOC exhibited high resistance to heterologous spike-mediated immunity (0% and 25% survival against SMEN_WA1_ and SMEN_Omicron_ vaccine) among the variants we tested in mice (Figure 4 and Figure 5, Table 1). This prompted us to explore if the SMEN vaccine presenting Beta spike can better elicit broadly protective humoral immune responses. To this end, we immunized groups of K18-hACE2 mice with either adjuvant or SMEN_Beta_ VLPs using the combined regimen, then challenged them with either the homologous Beta VOC or the heterologous WA1, Delta, or the Omicron variant (Figure 6A). As expected, the SMEN_Beta_ vaccine significantly reduced body weight loss and protected 100% of the mice against the homologous Beta VOC challenge, in contrast to the adjuvant-treated mice that succumbed to the infection. Furthermore, SMEN_Beta_-vaccinated mice showed 100% protection against lethal challenge with the heterologous WA1 or Delta variants, as well as Omicron, where the vaccine-mediated immunity significantly prevented body weight loss (Figure 6B). Evaluation of viral loads in the lung and brain tissues of SMEN_Beta_-vaccinated mice was also consistent with the survival and body weight analyses, showing significant reductions and indicating effective virologic control against all four tested variants (Figure 6C and Figure S6A). Furthermore, the SMEN_Beta_-vaccinated mice displayed a significant reduction in mRNA expression of inflammatory cytokines in both brain and lung tissues during challenge with both homologous or heterologous variants (Figure S6B,C). As was the case with SMEN_WA1_ and SMEN_Omicron_, SMEN_Beta_ elicited antibodies against individual proteins in VLPs and cross-reacted with heterologous spike proteins, indicating successful seroconversion (Figure S6D). To better understand the breadth of protection, we assessed the cross-reactive neutralization and Fc-signaling activity in the SMEN_Beta_-elicited sera before the challenge (Figure 6D,E and Figure S6E and Table 1). The sera had a total CRNI of 159, the highest among the different spike-presenting VLPs tested. Despite low CRNI against Delta VOC (10), mean IC50 values were still relatively low (1.83 µL of sera), which likely provided sufficient protection and reduction in disease burden against a lethal Delta challenge (Figure 6D, Table 1). SMEN_Beta_-elicited sera also demonstrated a high cross-reactive Fc-signaling index of 162, second only to SMEN_Omicron_-elicited sera (190) (Figure 6E, Table 1). These data suggest that the SMEN_Beta_ vaccine generated superior cross-reactive neutralizing and Fc-effector potent antibodies among the SMEN variants tested. Thus, the Beta VOC not only resisted immunity produced by other variants but also triggered a diverse and effective humoral immune response. Our study suggests that using the Beta variant spike as a base for vaccine development may lead to better protection against emerging SARS-CoV-2 variants.

**Table 1:** The potency of Neutralizing Responses Elicited by SMEN Vaccines Used in the Study and Their Efficacy in Reducing SARS-CoV-2-Induced Mortality and Disease.

SMEN Vaccine	Homologous/Cross-Neutralization (Mean IC50; µL of Serum)				Mortality			Disease Burden			
	WA1	Delta	Beta	Omicron	WA1	Delta	Beta	WA1	Delta	Beta	Omicron *
Vehicle	-	-	-	-	100% Death 6 dpi	100% Death 6–7 dpi	100% Death 6 dpi	100	100	100	100 *
SMEN_WA1_ (i.m.)	0.16	-	-	-	0%	-	-	4.59	-	-	-
SMEN_WA1_ (i.n.)	1.30	-	-	-	0%	-	-	1.24	-	-	-
SMEN_WA1_ (i.m. + i.n.)	0.05	0.79	3.91	3.51	0%	25% Delayed Death 8 dpi	100% Delayed Death 7–8 dpi	0.41	9.50	48.8	-
SMEN_Omicron_ (i.m. + i.n.)	2.20	6.09	3.14	0.40	50% Delayed Death 9–11 dpi	100% Delayed Death 8–9 dpi	75% Delayed Death 6–10 dpi	15.61	44.16	39.20	9.37 *
SMEN_Beta_ (i.m. + i.n.)	0.42	1.83	0.22	0.32	0%	0%	0%	1.08	3.79	0.91	20.54 *

- not tested; * Mortality was excluded from the disease burden calculation as Omicron VOC infection is non-lethal (see Section 2 for details).

## 4. Discussion

Here, we have demonstrated the potential of the SMEN VLP vaccine platform in eliciting robust cross-protective immune responses against SARS-CoV-2 variants in vivo using a stringent K18-hACE2 mouse model. By employing S proteins from the ancestral WA1 strain, Beta, and Omicron BA.1 variant, we evaluated the in vivo efficacy of SMEN VLPs as vaccines. Our results highlighted that the combined intranasal and intramuscular administration of the SMEN vaccine generated the most effective immune responses, leading to superior virologic control, reduced lung inflammation, and a lower overall disease burden. These data align with the growing body of evidence highlighting the importance of mucosal immunity in addition to peripheral stimulation for better prognosis following infection by respiratory pathogens, including SARS-CoV-2 and influenza [56,57,58,59].

The observed SMEN-mediated immune protection was predominantly antibody-mediated, which is consistent with findings from several other vaccine platforms against SARS-CoV-2 [33,60,61]. SMEN vaccine not only elicited antibodies against the spike protein but also against the M, E, and N proteins. We expect the immune response to the M, E, and N proteins to be consistent across all the analyzed SMEN VLPs and contribute to enhancing overall immunity. Antibodies targeting the N protein have the potential to clear infected cells through Fc-mediated effector functions, as the N protein was shown to be present on infected cell surfaces [62,63]. Additionally, antibodies against the E protein can potentially mitigate E-protein-mediated inflammasome activation by blocking interaction with TLR2 [64,65], contributing to an overall reduction in lung pathology. In addition to antibodies, the SMEN vaccine also elicited T cells. However, we found that vaccine-elicited CD4^+^ and CD8^+^ T cells played a minor role, as their depletion did not compromise protection from virus-induced mortality. Unlike mRNA and adenovirus-based vaccines, SMEN antigens are not expressed within cells but are instead presented exogenously to the immune system. Consequently, diminished CD8^+^ T cell responses are expected, except for the contributions arising from cross-presentation [66,67]. However, T cells did contribute to limiting virus dissemination to distal organs, such as the brain, and reducing inflammation in the lungs. Limiting virus dissemination has important consequences, including a reduction in the multi-organ pathology of COVID-19 [68]. In addition, reducing infection-induced inflammation is known to significantly benefit post-infection recovery and reduce lung pathology [34,69,70].

Notably, SMEN particles presenting Beta spikes elicited diverse and potent humoral immune responses in terms of neutralizing and Fc-signaling activity. The basis for the broad response remains unclear and requires further investigation. The Beta variant was the first VOC that arose mainly due to its capacity for immune evasion [71]. The Beta variant S protein has several key mutations, particularly in the receptor-bind domain (RBD) and the N-terminal domain (NTD), which significantly alter its antigenic profile. The RBD mutations K417N change the conformation of RBD, whereas E484K substantially enhances nAb escape, and N501Y enhances binding affinity to the ACE2 receptor. These alterations also allow Beta VOC to overcome vaccine and infection-induced immunity [71]. Additionally, Beta infection has been shown to trigger responses with significantly improved Fc cross-reactivity against global VOCs compared to those elicited by the original D614G variant or by vaccination with Ad26.COV2.S, suggesting that the infecting spike sequence significantly impacts Fc effector function [72]. The ability of the Beta variant Spike protein to elicit cross-reactive antibodies is further supported by the identification of public clonotypes, such as the VH1-58 clonotype, which targets the RBD ridge independent of VOC mutations, indicating a broader neutralizing capability [71]. We propose that vaccinating with an immune-evasive conformational state, such as Beta S, elicits antibodies that are more capable of adapting to common escape mutations, thereby generating a broader immune response. Thus, the unique properties of the Beta spike protein, as previously noted [71,72], may serve as a middle-ground antigenic template in the virus-host co-evolutionary landscape that provides balanced and broad immunity against both previous and future variants of concern (VOCs). In contrast, the significant antigenic shift and divergence seen in the Omicron variant spike likely led to the overall reduction in nAb-mediated immunity against the ancestral strain and previous VOCs while broadening the overall reactivity through non-nAb responses. This was evident in our study by the markedly high cross-reactive Fc-signaling index in the sera of SMEN_Omicron_-vaccinated mice.

Given the intense immune pressure elicited by the host, viral fusion glycoproteins display elaborate immune evasion strategies, including structural plasticity, sequence variability, conformational flexibility, and glycosylation of vulnerable epitopes [73]. One vaccine strategy to counter immune diversion is to lock fusion glycoproteins in the most vulnerable prefusion conformation [73,74,75]. This approach focuses on humoral immune responses to elicit antibodies that are predominantly neutralizing in nature to effectively block virus entry. This strategy was harnessed by various vaccine platforms with the strategic introduction of conformationally rigid proline residues in the S2 region of the spike protein to present the prefusion conformation as vaccine antigens during the COVID-19 pandemic [76]. Restricting conformational flexibility is of paramount importance in designing vaccines against the highly immune-evasive HIV-1 to elicit effective nAbs and remains one of the unachieved goals in HIV-1 vaccinology [77]. However, locking conformations may not necessarily enhance effective immune response for all fusion glycoproteins, as shown for the human cytomegalovirus type III fusion glycoprotein gB [78]. In the case of SARS-CoV-2, which is predominantly controlled through humoral immune responses, stabilizing specific conformations, while tremendously successful, might also limit the breadth of humoral immune responses. In this regard, the SMEN VLP platform has the advantage of presenting an array of membrane-embedded native conformations seen in virions that can potentially contribute to eliciting humoral immune responses to additional vulnerable conformations. Furthermore, the modular design of the vaccine platform enables the production of VLPs carrying spike proteins from multiple VOCs or combining SMEN particles with different spikes to enhance cross-reactivity. Overall, our study adds to the utility of the SMEN VLP platform as an additional vaccine option that can complement existing ones and potentially provide safer alternatives to boost acceptance rates. This versatility and broad-spectrum efficacy make SMEN VLPs a promising tool in the ongoing battle against COVID-19 and future emerging infectious diseases.

## Data Availability

All data supporting the findings of this study are available within the paper and its Supplementary Materials.

## Supplementary Materials

The following supporting information can be downloaded at: https://www.mdpi.com/article/10.3390/vaccines12091007/s1, Figure S1: Production and characterization of the SARS-CoV-2 virus-like particle (SMEN) vaccine and its impact on neutralizing antibody response, Figure S2: Passive transfer of SMEN-vaccine elicited sera provides immunity to naïve mice against lethal SARS-CoV-2 challenge, Figure S3: CD8^+^ and CD4^+^ T cells contribute to SMEN Vaccine-mediated protection by diminishing virus spread to the brain, Figure S4: SMEN_WA1_ Vaccine Shows Efficacy Against Delta and Offers Minimal Cross-Protection Against Beta VOC, Figure S5: SMEN_Omicron_ Offers Limited Cross-Protection Against Heterologous VOCs, Figure S6: SMEN_Beta_ Offers Broad Cross-Protection Against Heterologous VOCs.
