# Supplementary material for: Beta Spike-Presenting SARS-CoV-2 Virus-like Particle Vaccine Confers Broad Protection against Other VOCs in Mice
Source: Vaccines (Basel). 2024 Sep 2;12(9):1007. doi: 10.3390/vaccines12091007 (PMC11435481; doi:10.3390/vaccines12091007)
Supplement: Supplementary file 1 [file vaccines-12-01007-s001.zip › vaccines-3080016-supplementary.pdf]

vaccines

MDPI

Article

The Beta Spike-Presenting SARS-CoV-2 Virus-like Particle Vaccine Confers Broad Protection Against Other VOCs in Mice

Irfan Ullah [1], Kelly Symmes [1], Kadiatou Keita [2], Li Zhu [1], Michael W. Grunst [2], Wenwei Li [2], Walther Mothes [2], Priti Kumar [1], Pradeep D. Uchil [2,*]

[1] Department of Internal Medicine, Section of Infectious Diseases, Yale University School of Medicine, New Haven, CT 06520, USA; irfan.ullah@yale.edu (I.U.); kelly.symmes@yale.edu (K.S.); li.zhu@yale.edu (L.Z.); priti.kumar@yale.edu (P.K.)

[2] Department of Microbial Pathogenesis, Yale University School of Medicine, New Haven, CT 06510, USA;USA; k.keita@yale.edu (K.K.); mikey.grunst@yale.edu (M.W.G.); wenwei.li@yale.edu (W.L.); walther.mothes@yale.edu (W.M.)

* Correspondence: pradeep.uchil@yale.edu

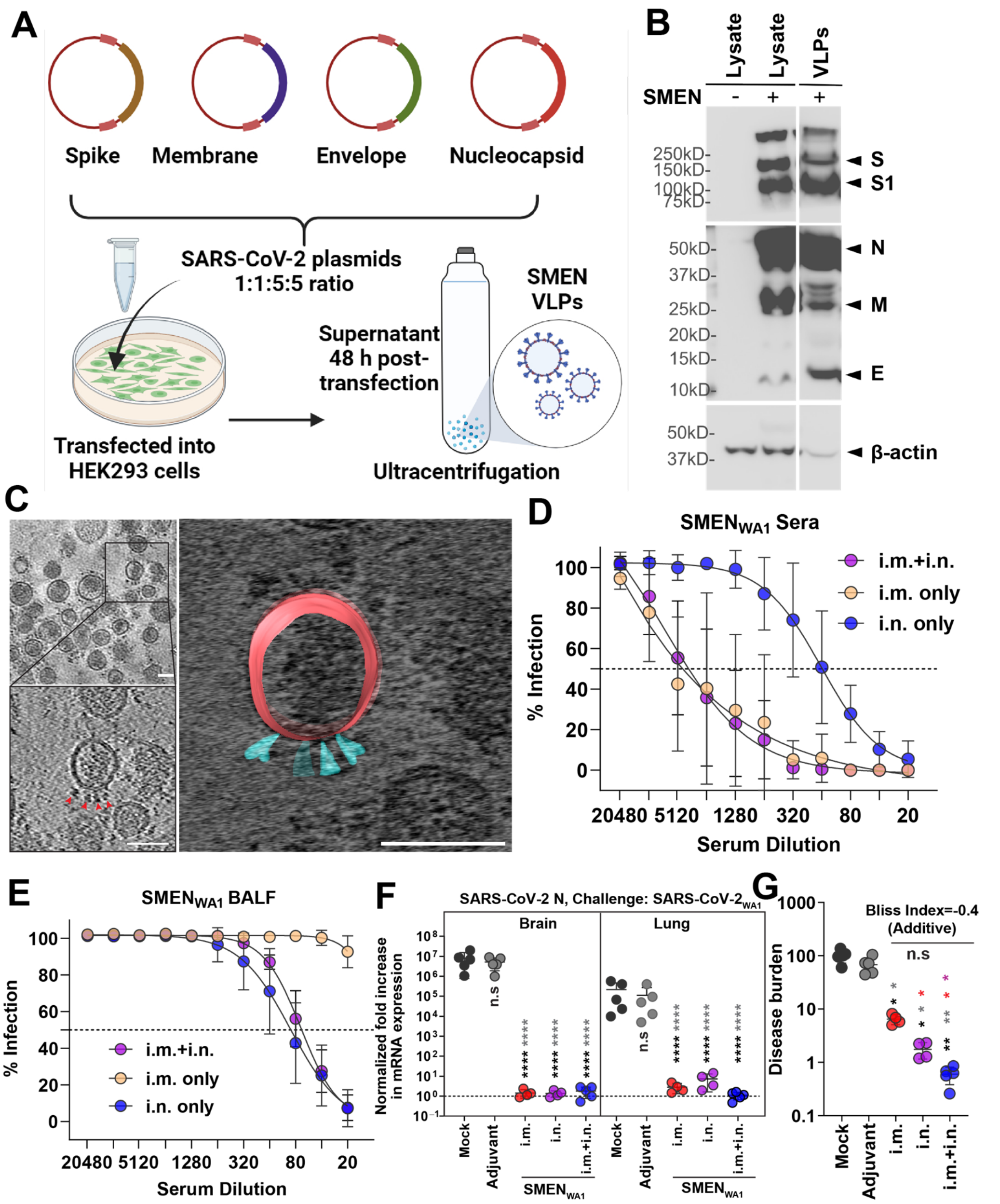

Figure S1. Production and characterization of SARS-CoV-2 virus-like particle (SMEN) vaccine and its impact on neutralizing antibody responses. (**A**) Schematic illustration of the experimental design for producing SMEN VLPs. Individual plasmids expressing the SARS-CoV-2 spike, membrane, envelope, and nucleocapsid proteins were transfected into HEK293 cells. VLPs were then collected and concentrated from culture supernatants. (**B**) Western blot analysis of producer cell lysates and purified SMEN VLPs probed with antibodies to strep tag as the expressed proteins had strep tags for analyses. Antibodies to beta-actin were used as control. (**C**) Cryo-electron microscopy (Cryo-EM) images of SARS-CoV-2 SMEN VLPs, demonstrating presence of spike protein on VLPs. Scale bar: 50 nm. (**D, E**) SARS-CoV-2WA1 neutralizing activity in sera and BALF from SMENWA1 vaccinated mice, harvested before challenge, using live virus for an experiment shown in Figure 1A. Sera or BALF samples were serially diluted (4-fold dilution) and pre-incubated with 200 PFU of SARS-CoV-2 WA1-nLuc for 30 minutes before infection. nLuc activity was measured 24 hpi. Virus-infected cells without sera or BALF were set at 100%. (**F**) Viral loads [SARS-CoV-2 Nucleocapsid (N) mRNA expression] in indicated organs from mice under specified treatment regimens. Data were normalized to *Gapdh* mRNA in the same sample

and that in non-infected mice after necropsy. (**G**) Multiparametric disease burden and Bliss index estimation (see Method details) for indicated groups of mice to compare efficacies of vaccination regimens. Bliss index score of -10 to 10 is considered as additive effect. Grouped data in (F) were analyzed by 2-way ANOVA followed by Tukey's multiple comparison tests and data in (G) was analyzed by pairwise non-parametric Mann-Whitney test. Statistical significance for the groups comparisons to mock are shown in black, with adjuvant are shown in light dark, with $SMEN_{WA1}$ i.m. are shown as red, with $SMEN_{WA1}$ i.n. are shown as magenta. *, $p < 0.05$; **, $p < 0.01$; ns, not significant; Mean values ± SD are depicted.

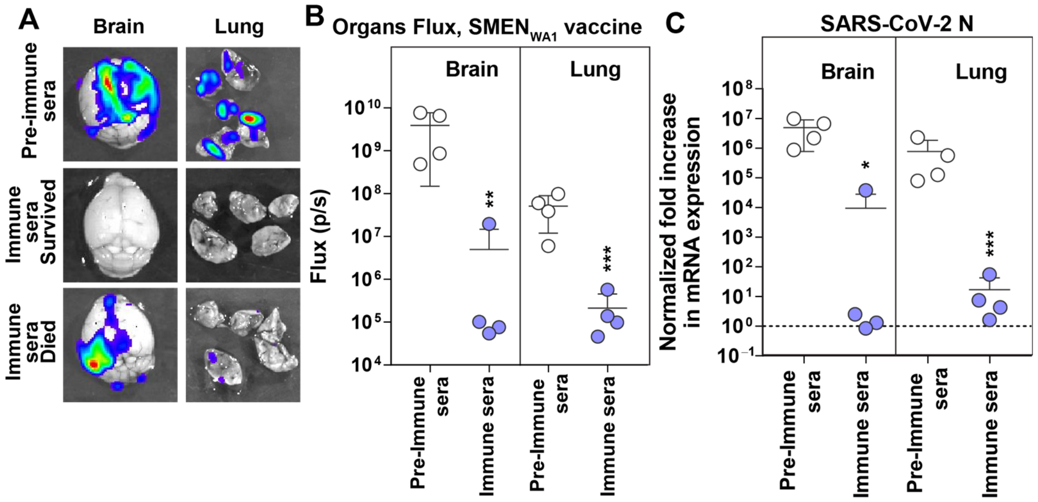

Figure S2. Passive Transfer of SMEN-vaccine Elicited Sera Provides Immunity to Naïve Mice Against Lethal SARS-CoV-2 Challenge. (**A, B**) *Ex vivo* imaging of indicated organs and quantification of nLuc signals as flux (photons/sec) after necropsy in K18-hACE2 mice for an experiment shown in Figure 2A. (**C**) Viral loads (SARS-CoV-2 Nucleocapsid mRNA expression) in indicated organs from mice under specified treatment regimens at the time of death or at 14 dpi for surviving mice. Data were normalized to *Gapdh* mRNA in the same sample and that in non-infected mice after necropsy. Grouped data in (B and C) were analyzed by 2-way ANOVA followed by Tukey's multiple comparison tests. Statistical significance for the groups comparisons to pre-immune sera are shown in black ∗, $p < 0.05$; ∗∗, $p < 0.01$; ∗∗∗, $p < 0.001$; ∗∗∗∗, $p < 0.0001$; ns, not significant; Mean values ± SD are depicted.

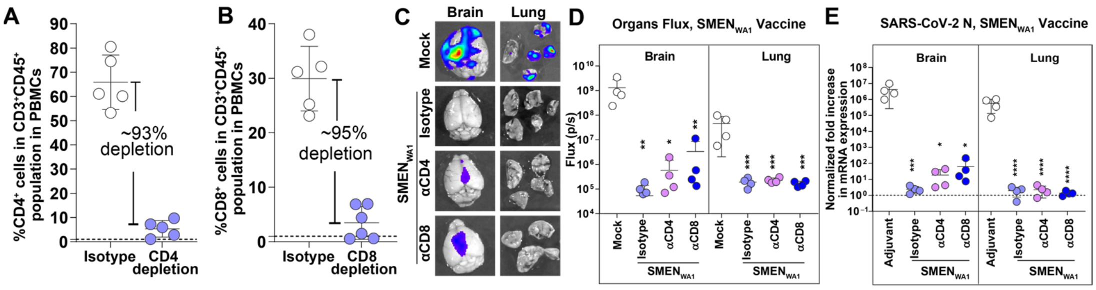

Figure S3. CD8⁺ and CD4⁺ T cells contribute to SMEN Vaccine-mediated protection by diminishing virus spread to the brain (**A**) Comparison of percent $CD4^+$ and $CD8^+$ T cell populations in the blood after i.p. administration of isotype, anti-CD4 or anti-CD8*α* T cell depleting antibodies in SMEN-vaccinated K18-hACE2 mice (n=4; two experiments). (**C, D**) *Ex vivo* imaging of indicated organs and quantification of nLuc signals as flux (photons/sec) after necropsy in K18-hACE2 mice for an experiment shown in Figure 3A. (**E**) Viral loads (SARS-CoV-2 Nucleocapsid, N mRNA) in indicated organs from mice under specified treatment regimens at the time of death or at 14 dpi for surviving mice. Data were normalized to *Gapdh* mRNA in the same sample and that in non-infected mice after necropsy. Grouped data in (D, E) were analyzed by 2-way ANOVA followed by Tukey's multiple comparison tests. Statistical significance for the groups comparisons to adjuvant are shown in black, with $CD4^+$ T-cells depleted group are shown as magenta and with $CD8^+$ T-cells depleted group are shown as blue. ∗, $p < 0.05$; ∗∗, $p < 0.01$; ∗∗∗, $p < 0.001$; ∗∗∗∗, $p < 0.0001$; ns, not significant; Mean values ± SD are depicted.

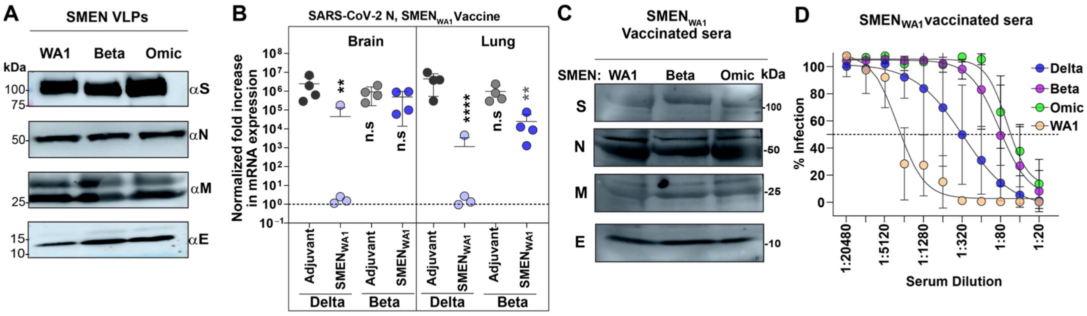

Figure S4. SMEN$_{WA1}$ Vaccine Shows Efficacy Against Delta and Offers Minimal Cross-Protection Against Beta VOC. (**A**) Characterization of SMEN VLPs displaying spikes from the specified VOCs, analyzed by Western blotting and probed with antibodies to spike, nucleocapsid, membrane and envelope proteins. (**B**)Viral loads (SARS-CoV-2 Nucleocapsid, N mRNA) in indicated organs from mice under specified treatment regimens at the time of death or at 22 dpi for surviving mice. Data were normalized to *Gapdh* mRNA in the same sample and that in non-infected mice after necropsy. Statistical significance in (**B**) for the group comparisons to adjuvant with Delta VOC infected mice are shown in black, to adjuvant with beta VOC infected mice are shown in grey. *, $p < 0.05$; **, $p < 0.01$; ***, $p < 0.001$; ****, $p < 0.0001$; ns, not significant; Mean values ± SD are depicted. (**C**) Western blot analyses of indicated SMEN VLPs probed with sera from SMEN$_{WA1}$ vaccinated mouse **(D)** Live virus neutralization assay for sera from SMEN$_{WA1}$ vaccinated mice harvested before challenge against WA1, Delta and Beta, Omicron variants. Sera samples were serially diluted (4-fold dilution starting at 1:10 dilution) and pre-incubated with 150 PFU of SARS-CoV-2 WA1-nLuc, Delta-nLuc, Beta-nLuc and 200 PFU of Omicron-nLuc for 30 minutes before infection. nLuc activity was measured 24 hpi. Virus-infected cells without sera were set at 100%.

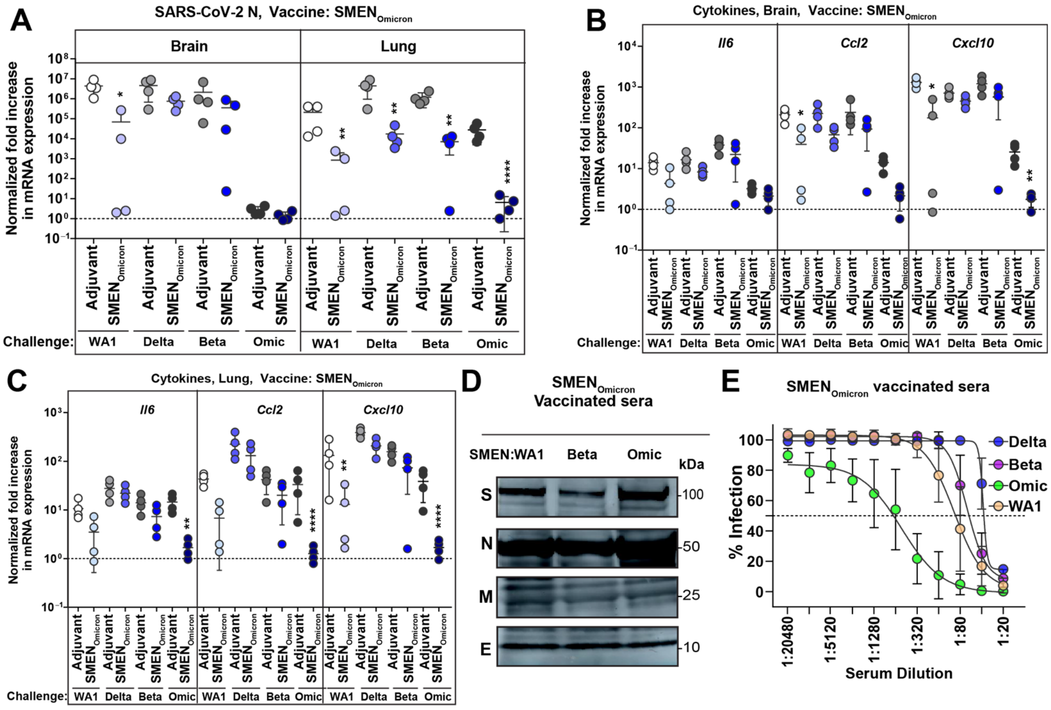

Figure S5. **SMEN$_{Omicron}$ Offers Limited Cross-Protection Against Heterologous VOCs.** (**A**) Viral loads (SARS-CoV-2 Nucleocapsid, N mRNA) in indicated organs from mice under specified treatment regimens at the time of death or at 16 dpi for surviving mice for an experiment as in Figure 6A. (**B, C**) Fold changes in indicated cytokine mRNA expression in brain and lung tissues of mice under specified treatment regimens after necropsy upon death or at 16 dpi in the surviving animals. Data were normalized to *Gapdh* mRNA in the same sample and that in non-infected mice after necropsy. (**D**) Western blot analyses of indicated SMEN VLPs probed with sera from SMEN$_{Omicron}$ vaccinated mouse (**E**) Live virus neutralization assay for sera from SMEN$_{Omicron}$ vaccinated mice (n=4, each dot represents one mouse) harvested before challenge, against WA1, Delta and Beta, Omicron variants. Sera samples were serially diluted (4-fold dilution starting at 1:10 dilution) and pre-incubated with 150 PFU of SARS-CoV-2 WA1-nLuc, Delta-nLuc, Beta-nLuc and 200 PFU of Omicron-nLuc for 30 minutes before infection. nLuc activity was measured 24 hpi. Virus-infected cells without sera were set at 100%. Statistical significance for the group comparisons to adjuvant with each cohort are shown in black. *, $p < 0.05$; **, $p < 0.01$; ****, $p < 0.0001$; ***, $p < 0.001$; ns, not significant; Mean values ± SD are depicted.

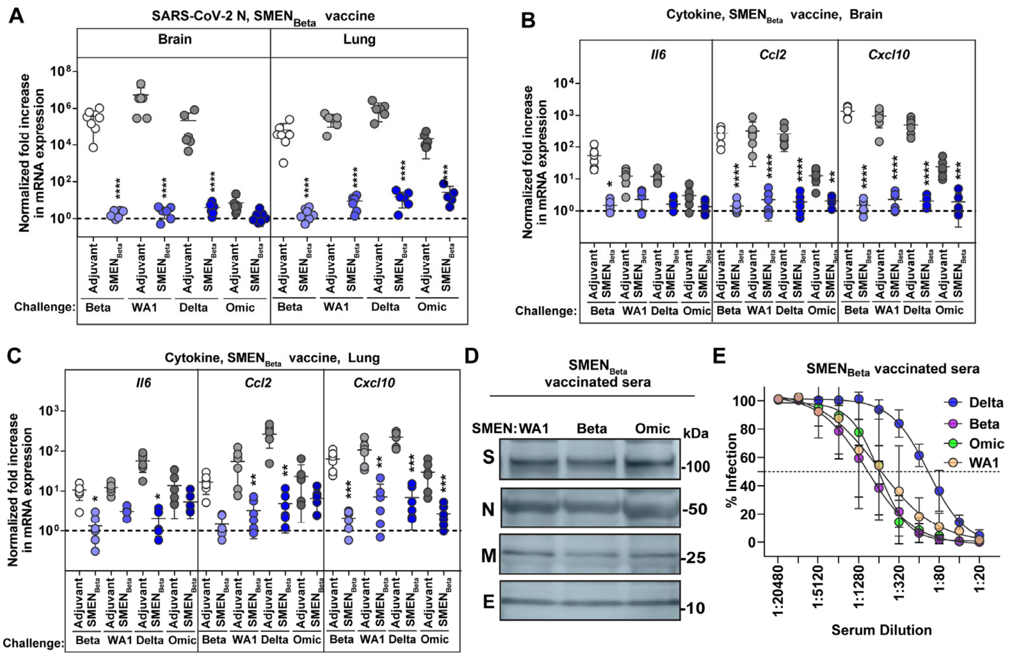

Figure S6. SMENBeta Offers Broad Cross-Protection Against Heterologous VOCs. (**A**) Viral loads (SARS-CoV-2 Nucleocapsid, N mRNA) in indicated organs from mice under specified treatment regimens at the time of death or at 16 dpi for surviving mice for an experiment as in Figure 6A. (**B, C**) Fold changes in indicated cytokine mRNA expression in brain and lung tissues of mice under specified treatment regimens after necropsy upon death or at 16 dpi in the surviving animals. Data were normalized to *Gapdh* mRNA in the same sample and that in non-infected mice after necropsy. (**D**) Western blot analyses of indicated SMEN VLPs probed with sera from SMEN*Beta* vaccinated mouse (**E**) Live virus neutralization assay for sera from SMENBeta vaccinated mice (n=4, each dot represents one mouse) harvested before challenge, against WA1, Delta and Beta, Omicron variants. Sera samples were serially diluted (4-fold dilution starting at 1:10 dilution) and pre-incubated with 150 PFU of SARS-CoV-2 WA1-nLuc, Delta-nLuc, Beta-nLuc and 200 PFU of Omicron-nLuc for 30 minutes before infection. nLuc activity was measured 24 hpi. Virus-infected cells without sera were set at 100%. Statistical significance for the group comparisons to adjuvant with each cohort are shown in black. *, $p < 0.05$; **, $p < 0.01$; ****, $p < 0.0001$; ***, $p < 0.001$; ns, not significant; Mean values ± SD are depicted.

Disclaimer/Publisher's Note: The statements, opinions and data contained in all publications are solely those of the individual author(s) and contributor(s) and not of MDPI and/or the editor(s). MDPI and/or the editor(s) disclaim responsibility for any injury to people or property resulting from any ideas, methods, instructions or products referred to in the content.
